# A COVID-19 risk score combining chest CT radiomics and clinical characteristics to differentiate COVID-19 pneumonia from other viral pneumonias

**DOI:** 10.18632/aging.202735

**Published:** 2021-03-13

**Authors:** Zuhua Chen, Xiadong Li, Jiawei Li, Shirong Zhang, Pengfei Zhou, Xin Yu, Yao Ren, Jiahao Wang, Lidan Zhang, Yunjiang Li, Baoliang Wu, Yanchun Hou, Ke Zhang, Rongjun Tang, Yongguang Liu, Zhongxian Ding, Bin Yang, Qinghua Deng, Qin Lin, Ke Nie, Zhaobin Cai, Shenglin Ma, Yu Kuang

**Affiliations:** 1 Department of Radiology, Hangzhou Xixi Hospital, Hangzhou 310000, Zhejiang, China; 2 Department of Radiology, Hangzhou 6th People’s Hospital, the Affiliated Hospital of Zhejiang Chinese Medical University, Hangzhou 310000, Zhejiang, China; 3 Department of Radiation Oncology, Hangzhou Cancer Hospital, Zhejiang University Cancer Centre, Hangzhou First People’s Hospital Group, Hangzhou 310000, Zhejiang, China; 4 Department of Radiation Oncology, Affiliated Hangzhou First People’s Hospital, Zhejiang University School of Medicine, Hangzhou 310000, Zhejiang, China; 5 Department of Radiology, The Fourth Clinical Medical College, Zhejiang Chinese Medical University, Hangzhou 310000, Zhejiang, China; 6 Department of Radiation Oncology, Rutgers Cancer Institute of New Jersey, Rutgers University, New Brunswick, NJ 07097, USA; 7 Medical Physics Program, University of Nevada, Las Vegas, NV 89154, USA; 8 Department of Radiation Oncology, Xiamen Cancer Hospital, The First Affiliated Hospital of Xiamen University, Teaching Hospital of Fujian Medical University, Xiamen 361003, Fujian, China

**Keywords:** coronavirus disease 2019, COVID-19, severe acute respiratory syndrome coronavirus 2, chest CT, radiomics, nomogram

## Abstract

With the continued transmission of severe acute respiratory syndrome coronavirus 2 (SARS-CoV-2) throughout the world, identification of highly suspected COVID-19 patients remains an urgent priority. In this study, we developed and validated COVID-19 risk scores to identify patients with COVID-19. In this study, for patient-wise analysis, three signatures, including the risk score using radiomic features only, the risk score using clinical factors only, and the risk score combining radiomic features and clinical variables, show an excellent performance in differentiating COVID-19 from other viral-induced pneumonias in the validation set. For lesion-wise analysis, the risk score using three radiomic features only also achieved an excellent AUC value. In contrast, the performance of 130 radiologists based on the chest CT images alone without the clinical characteristics included was moderate as compared to the risk scores developed. The risk scores depicting the correlation of CT radiomics and clinical factors with COVID-19 could be used to accurately identify patients with COVID-19, which would have clinically translatable diagnostic and therapeutic implications from a precision medicine perspective.

## INTRODUCTION

The novel severe acute respiratory syndrome coronavirus 2 (SARS-CoV-2) has been identified as the cause of coronavirus disease 2019 (COVID-19) in Wuhan, Hubei Province, China in late 2019 [1]. It spread rapidly, resulting in a global pandemic, with over 23,342,798 confirmed cases and 807,383 deaths globally as of August, 2020 [2]. COVID-19 developed through person-to-person spread of SARS-CoV-2 via respiratory droplets is associated with adverse outcomes, and increased short- and long-term morbidity and mortality [1]. The identification of suspected patients with COVID-19 is urgently needed so that we can evaluate patients at greater risk and/or more vulnerable to COVID-19 and facilitate appropriate clinical decision making for earlier quarantine and interventions that could minimize the severity of COVID-19, thus substantially improving patient outcome.

Currently, the standard for the diagnosis of COVID-19 is the use of the reverse transcription polymerase chain reaction (RT-PCR) to detect SARS-CoV-2 in lower throat respiratory tract secretions, sputum, swabs, or blood samples [3]. However, the sensitivity of the RT-PCR varies within a range of 60–71% because its accuracy could be compromised by the quality of the RT-PCR kit, the varying lowest limit of detection (LOD) of virus RNA copies per mL with the kits of different vendors, the quality and location of specimens collected (upper vs. lower respiratory tract), the low viral load in test specimens collected, and/or sampling timing (different phases of the disease), thus easily leading to false negative results [3, 4].

Recently, the European Society for Radiotherapy and Oncology (ESTRO) and the American Society for Radiation Oncology (ASTRO) jointly issued an ESTRO-ASTRO consensus statement to recommend the use of simulation-CT in clinical practice as a COVID-19 screening tool during the SARS-CoV-2 pandemic [5]. The consensus statement suggests that the CT imaging techniques used in radiotherapy are a potential screening opportunity and may be an added value to identify asymptomatic COVID-19 patients that are not identified by standard screening in hospitals (e.g., temperature screening and questions regarding COVID-19-related symptoms) [5]. The consensus was based on the fact that studies using CT imaging have identified patients with COVID-19 with negative RT-PCR results [6, 7]. In particular, thoracic CT screening allows early diagnosis of COVID-19, when patients are still in the asymptomatic phase [5–8].

Chest radiography and CT imaging have a sensitivity of 56–98% to identify suspected patients before the occurrence of positive RT-PCR detection results as well as use to assess disease extent and follow-up [4]. The principal CT manifestations include ground-glass opacification (GGO) with or without consolidative abnormalities and a bilateral, peripheral, and diffuse distribution with or without an involvement of the lower lobes [4, 9]. Especially, asymptomatic patients with initially negative RT-PCR results also showed early CT changes [9].

However, the identification of CT manifestations highly relies on radiologists’ clinical experience due to the qualitative CT features used, which might pose a challenge to resource-limited clinics with health care disparities for COVID-19 diagnosis. Meanwhile, COVID-19 shares similar manifestations with severe acute respiratory syndrome (SARS), Middle East respiratory syndrome (MERS), and other viral pneumonias in the images of qualitative CT, thus significantly reducing the specificity of qualitative CT for COVID-19 detection [10]. As such, chest CT may be helpful in making the diagnosis, but no finding can completely confirm or exclude the possibility of COVID-19 without including other clinical characteristics due to the extremely low specificity of 25% of the chest CT alone for diagnostic purposes [4]. These aspects of qualitative CT emphasize limitations of the current imaging model for diagnosing COVID-19 before the occurrence of its clinical symptoms and have compelled radiologists to call for new, imaging-based methods to answer this critical clinical question.

Digital biopsy techniques have evolved to use high-throughput processes to extract quantifiable radiomic features from medical images and have the potential to facilitate disease characterization and assessment. The aim of this study was to develop and validate clinically translatable COVID-19 risk scores encompassing chest CT radiomics with or without clinical characteristics included for distinguishing COVID-19 from other viral pneumonia. As a reference, we also compared the prediction performance of the risk scores with that of 130 well-experienced radiologists from the epicenters of COVID-19 outbreak and non-epicenters in China as well as that of other machine learning methods in this study. The risk scores integrating the spatial information derived from chest CT radiomic features and/or clinical characteristics could better characterize the SARS-CoV-2 infection landscape, which still significantly overlaps with other virus-induced pneumonias in visual inspection of CT manifestations.

## RESULTS

### Patient characteristics

**Figure 1:**
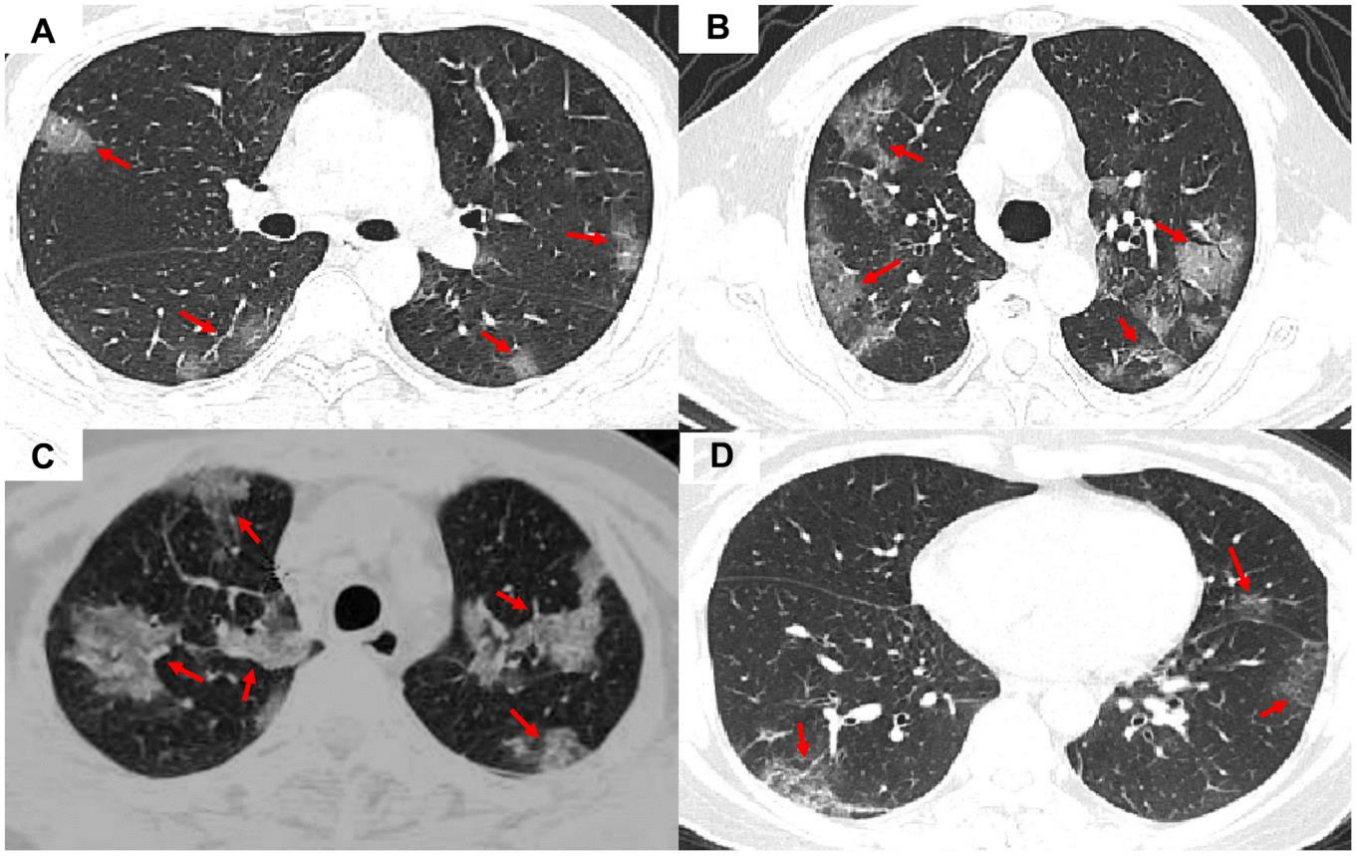
**Representative images of COVID-19 pneumonia, adenovirus pneumonia, cytomegalovirus pneumonia, and influenza virus pneumonia.** (**A**) A transverse CT image from a 35-year-old man with adenovirus pneumonia showing bilateral ground-glass opacities in the upper lobes with a rounded morphology (arrows). (**B**) COVID-19: A transverse CT image from a 57-year-old man with COVID-19 showing more limited ground-glass opacities in the bilateral upper lobes with an elliptical morphology (arrows). (**C**) A transverse CT image obtained in a 45-year-old female with cytomegalovirus pneumonia showing bilateral ground-glass and burr-like, denser, and less transparent distribution (arrows). (**D**) A transverse CT image of a 61-year-old man diagnosed with influenza virus pneumonia showing bilateral ground-glass opacities in the upper lobes (arrows).

The clinical characteristics of patient data used are shown in Table 1. The COVID-19 patients had significantly higher lesion numbers, CK-MB activity, LDH activity, and bilateral, peripheral, or mixed central and peripheral pulmonary distribution than the non-COVID-19 viral-induced pneumonia patients. Most of the symptoms, laboratory results, and CT manifestations had no significant differences between COVID-19 and non-COVID-19 patients (Table 1). Representative images of COVID-19 pneumonia, adenovirus pneumonia, cytomegalovirus pneumonia, and influenza virus pneumonia are shown in Figure 1.

**Table 1:** Clinical characteristics of the COVID-19 and non-COVID-19 (viral-induced pneumonias) patient cohorts.

**Characteristics**	**COVID-19 patients (n = 108)**	**Non-COVID-19 patients (viral-induced pneumonias) (n = 77)**	***P*-value**
Age, years			
>50	41	27	0.002
≤50	67	50	0.234
Lesion number			
1 ≤ *n* < 3	14	72	<0.001
3 ≤ *n* < 5	12	5	0.729
5 ≤ *n* < 10	64	0	<0.001
10 ≤ *n*	18	0	<0.001
Sex			
Male	44	28	0.322
Female	64	49	0.876
Epidemiologic contact			
Travel history to Hubei Province, China^ξ^	12	—	—
Travel history to Wenzhou city, Zhejiang Province, China^ξ^	22	—	—
Unknown exposure	74	—	—
Symptoms			
Fever	89	57	0.437
Dyspnea	51	55	0.051
Chest tightness	17	14	0.121
Cough	67	75	0.532
Sputum	23	54	0.067
Rhinorrhea	37	65	0.213
Asymptomatic	17^*^	2	—
Laboratory results			
D-dimers, mg/L	0.51 ± 0.44	0.52 ± 0.34	0.897
C-reactive protein, mg/L	12.32 ± 18.7	7.49 ± 14.27	0.055
White blood cells, 10^9^/L	3.31 ± 2.13	3.58 ± 1.94	0.112
Creatine kinase isoenzyme, μg/L	9.12 ± 5.56	13.93 ± 5.69	<0.001
Lactate dehydrogenase, U/L	245.91 ± 75.35	167.35 ± 42.88	<0.001
CT manifestations			
Location			
Unilateral	1	75	<0.001
Bilateral	107	2	<0.001
Distribution			
Central	1	3	0.233
Peripheral	73	72	0.191
Central + peripheral	34	2	0.013
Main features			
Ground-glass opacity	67	43	0.278
Consolidation	11	9	0.055
Linear opacity	23	12	0.231
Mixed type	7	13	0.101
Interstitial change			
Septal thickening	37	25	0.062
Fine reticular opacity	11	39	0.012
Other features			
Vascular thickening	17	39	0.054
Crazy-paving pattern	45	39	0.123
Pleural thickening	13	2	0.053
Pleural effusion	0	0	—

^ξ^Two epicenters of the COVID-19 outbreak in China.

*These were tested as close contacts with confirmed COVID-19 patients.

### Human diagnosis of COVID-19

Supplementary Figure 5 shows the geographic distribution of 130 radiologists from 10 provinces in China, including Hubei. The performance of the 130 radiologists based on the chest CT images only (without providing patients’ clinical information and laboratory results) was moderate due to the overlap of CT manifestations between COVID-19 lesions and non-COVID-19 viral pneumonia lesions using a supervised human learning format (Table 2). Notably, the radiologists from Hubei Province, China, the epicenter of the COVID-19 outbreak in China, had a better performance than the radiologists from outside of Hubei Province (*P* < 0.05).

**Table 2:** Performance of the radiologists to diagnose COVID-19 from chest CT images.

	**Performance**	Radiologists from Hubei Province, China^*^ (n = 40)	**Radiologists from outside of Hubei Province, China (n = 90)**	***P*-value**
Assistant attending radiologists	*n*	13	42	—
	Average time for reviewing each CT image (sec)	3.2 ± 2.3	3.3 ± 2.1	0.132
	Precision	0.49	0.3	0.153
	Recall	0.30	0.23	0.121
	Specificity	0.57	0.39	0.058
	F1	0.37	0.28	0.131
	Accuracy	0.41	0.30	0.154
Associate attending radiologists	*n*	15	34	—
	Average time for reviewing each CT image (sec)	3.3 ± 2.6	3.6 ± 2.8	0.053
	Precision	0.49	0.35	0.002
	Recall	0.29	0.24	0.042
	Specificity	0.57	0.39	0.051
	F1	0.37	0.28	0.032
	Accuracy	0.41	0.30	0.021
Attending radiologists	*n*	12	14	—
	Average time for reviewing each CT image (sec)	3.1 ± 2.4	3.2 ± 2.9	0.129
	Precision	0.47	0.34	0.215
	Recall	0.29	0.22	0.055
	Specificity	0.54	0.40	0.067
	F1	0.35	0.26	0.042
	Accuracy	0.39	0.29	0.102
Overall	Average time for reviewing each CT image (sec)	3.2 ± 0.1	3.4 ± 0.2	0.054
	Precision	0.48	0.35	<0.001
	Recall	0.29	0.23	0.027
	Specificity	0.56	0.39	0.002
	F1	0.36	0.28	0.034
	Accuracy	0.41	0.30	0.029

^*^Hubei Province was the epicenter of the COVID-19 outbreak in China.

### Patient-based risk scores

**Figure 2:**
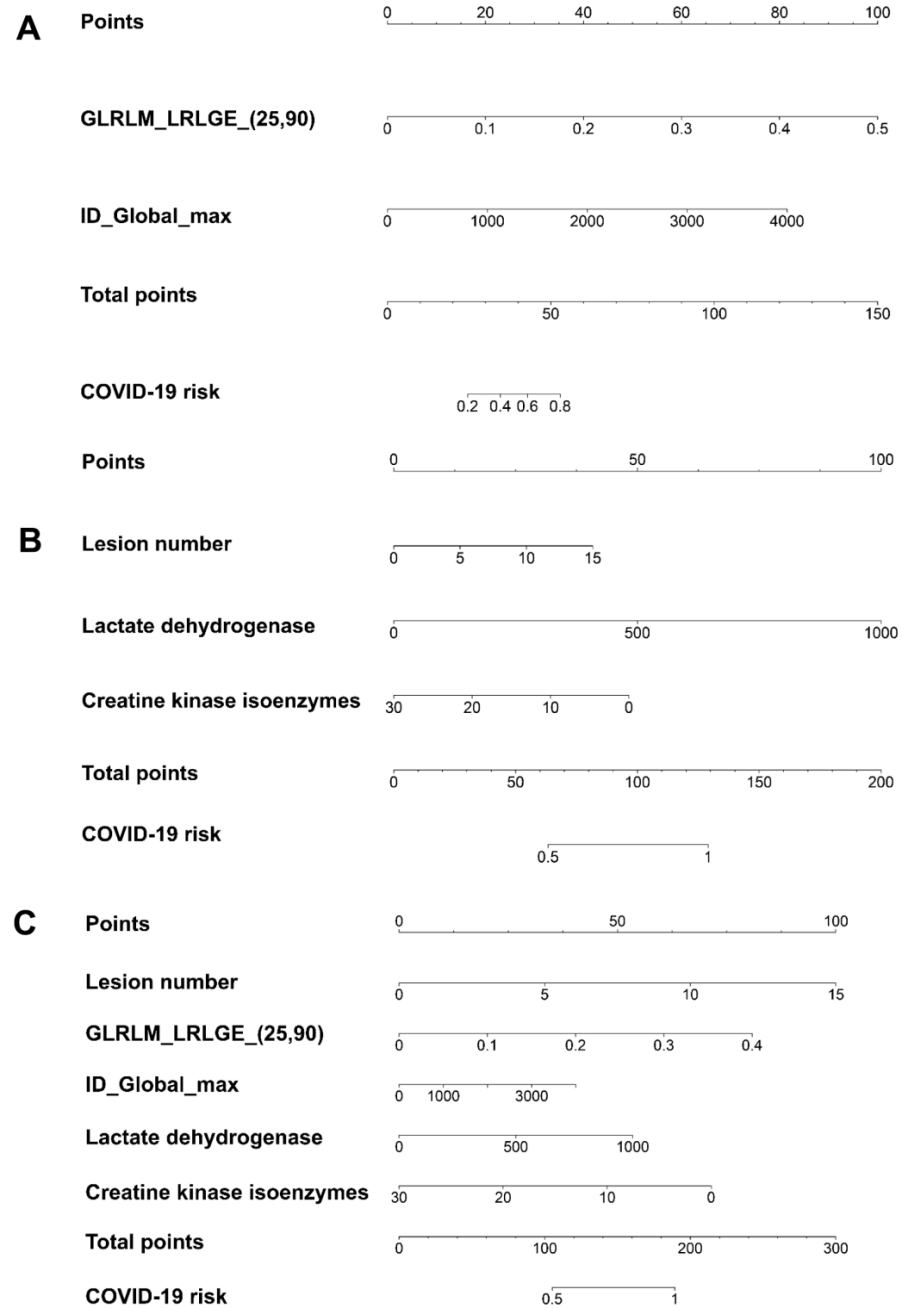
**The patient-based COVID-19 risk scores demonstrated by nomograms.** (**A**) The risk score using radiomic features only. (**B**) The risk score using clinical factors only. (**C**) The risk score combining radiomic features and clinical factors. GLRLM_LRLGE_(25, 90) represents the radiomic feature long run low gray-level emphasis, which describes the distribution of the long homogeneous runs with low gray-levels within the image. The numbers in the bracket represents the parameters used to calculate that particular radiomic feature. The parameters of 25 and 90 in GLRLM_LRLGE represent the binary mask in 2.5D and 90 degrees, which describes that the GLRLM was computed in 2D slice by slice; then, the occurrence of run length from 90 degrees from all 2D image slices was summed. ID_Global_max represents the radiomic feature intensity direct global max, which describes that the binary mask was preprocessed for the features derived directly from the image intensity. The binary mask in ID_Global_max can be modified through intensity thresholding, by binary erosion, and using only the binary slice with the maximum area. The unit for lactate dehydrogenase is U/L. The unit for creatine kinase isoenzymes is μg/L. Supplementary Appendix 2 explains how to use the nomograms.

**Figure 3:**
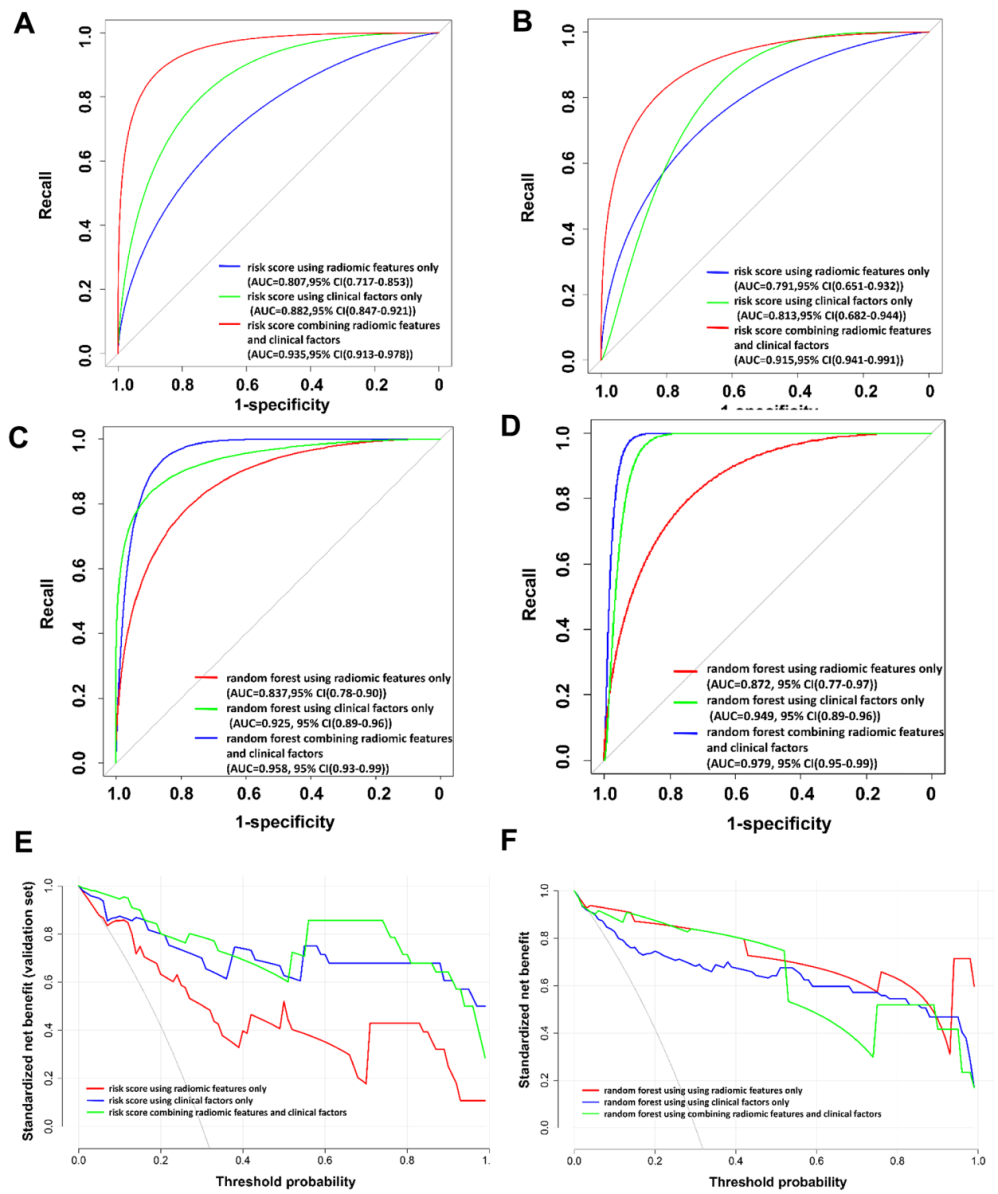
**The receiver operating characteristic (ROC) curves and the decision curve analysis (DCA) for the patient-based risk scores and random forest models.** (**A**) ROC curve for patient-based risk scores in the training set. (**B**) ROC curve for patient-based risk scores in the validation set. (**C**) ROC curve for patient-based random forest models in the training set. (**D**) ROC curve for patient-based random forest models in the validation set. (**E**) DCA for patient-based risk scores in the validation set. (**F**) DCA for patient-based random forest models in the validation set. In (**E**) and (**F**), the *x*-axis of the decision curve is the threshold of the predicted probability using the risk score to classify COVID-19 and non-COVID-19 patients. The *y*-axis shows the clinical decision net benefit for patients based on the classification result in this threshold. The decision curves of the treat-all scheme (the monotonically decreasing dash-line curve in the figure) and the treat-none scheme (the line when *x* equals zero) are used as references in the DCA. In this study, the treat-all scheme assumes that all the patients had COVID-19; the treat-none scheme assumes that none of the patients had COVID-19. Abbreviations: AUC, area under the ROC curve; 95% CI, 95% confidence interval.

The patient-based risk scores using radiomic features only, clinical factors only, and a combination of radiomic features and clinical factors are shown in Figure 2A–2C and Equations (2)–(4), respectively. The utility of the risk scores achieved area under the receiver operating characteristic curve (AUC) values of 0.791 (95% confidence interval [CI]: 0651–0.932), 0.813 (95% CI: 0.682–0.944), and 0.915 (95% CI: 0.841–0.991), respectively, in the validation set (Table 3 and Figure 3), suggesting a high performance of COVID-19 classification using the COVID-19 risk scores.

**Table 3:** The classification performance using patient-based COVID-19 risk scores and random forest models.

**Signature**	**AUC**	**Precision**	**Recall**	**Specificity**	**F1 score**	**Accuracy**	**Delong test for AUC values**
**Training set**							
COVID-19 risk score using radiomic features only	0.807 (0.717–0.853)	0.823 (0.703–855)	0.792 (0.711–0.834)	0.843. (0.781–0.867)	0.807 (0.723–0.897)	0.811 (0.703–0.966)	Z = 7.241, *P* = 0.000
COVID-19 risk score using clinical variables only	0.882 (0.847–0.921)	0.877 (0.712–0.903)	0.897 (0.784–0.922)	0.901 (0.879–0.944)	0.887 (0.813–0.922)	0.892 (0.824–0.927)	
COVID-19 risk score using combined radiomic and clinical variables	0.935 (0.913–0.978)	0.902 (0.878–0.966)	0.942 (0.807–0.989)	0.921 (0.893–0,964)	0.899 (0.812–0.962)	0.923 (0.879–0.978)	
Random forest using radiomic features only	0.837 (0.775–0.901)	0.712 (0.645–0,834)	0.896 (0.812–0.934)	0.877 (0.812–0.921)	0.793 (0.743–0.854)	0.845 (0.798–0.939)	Z = 8.574, *P* = 0.000
Random forest using clinical variables only	0.925 (0.892–0.963)	0.867 (0.772–0.934)	0.914 (0.854–0.963)	0.937 (0.879–0.987)	0.890 (0.807–0.919)	0.955 (0.913–0.977)	
Random forest using radiomic and clinical variables	0.958 (0.911–0.989)	0.886 (0.719–0.968)	0.934 (0.812–0.977)	0.954 (0.903–0.987)	0.909 (0.855–0.950)	0.966 (0.923–0.989)	
**Validation set**							Z = 9.307, *P* = 0.000
COVID-19 risk score using radiomic features only	0.791 (0.651–0.932)	0.804 (0.723–0.,902)	0.733 (0.693–0.854)	0.822 (0.734–0.876)	0.767 (0.717–0.856)	0.797 (0.701–0.892)	
COVID-19 risk score using clinical variables only	0.813 (0.682–0.944)	0.821 (0.721–0.876)	0.934 (0.877–0.965)	0.917 (0.832–0.989)	0.874 (0.793–0.941)	0.882 (0.769–0.923)	
COVID-19 risk score using combined radiomic and clinical variables	0.915 (0.841–0.991)	0.855 (0.744–0.913)	0.945 (0.897–0.988)	0.934 (0.899–0.989)	0.898 (0.844–0.953)	0.919 (0.87–0.955)	
Random forest using radiomic features only	0.872 (0.771–0.973)	0.809 (0.723–0.881)	0.913 (0.856–0.956)	0.896 (0.859–0.931)	0.858 (0.739–0.907)	0.868 (0.792–0.899)	Z = 7.896, *P* = 0.000
Random forest using clinical variables only	0.949 (0.894–0.956)	0.902 (0.843–0.977)	0.965 (0.913–0.998)	0.967 (0.943–0.997)	0.932 (0.899–0.956)	0.956 (0.933–0.979)	
Random forest using radiomic and clinical variables	0.979 (0.949–0.997)	0.943 (0.879–0.987)	0.987 (0.897–0.999)	0.934 (0.917–0.986)	0.964 (0.889–0.981)	0.963 (0.892–0.992)	

The values in the brackets represent the 95% confidence interval.

Abbreviations: AUC, area under the ROC curve.

$$\begin{array}{l} \text{T}\text{h}\text{e}\;\text{p}\text{a}\text{t}\text{i}\text{e}\text{n}\text{t}-\text{b}\text{a}\text{s}\text{e}\text{d}\;\text{r}\text{i}\text{s}\text{k}\;\text{s}\text{c}\text{o}\text{r}\text{e}\;\text{u}\text{s}\text{i}\text{n}\text{g}\;\text{r}\text{a}\text{d}\text{i}\text{o}\text{m}\text{i}\text{c}\;\text{f}\text{e}\text{a}\text{t}\text{u}\text{r}\text{e}\text{s}\;\text{o}\text{n}\text{l}\text{y}\\ \;\;\;\;\;\;\;\;\;\;\;\;\;\;\;\;\;\;=-3.785+19.563\times \;\text{G}\text{L}\text{R}\text{L}\text{M}\text{\_}\text{L}\text{R}\text{L}\text{G}\text{E}\text{\_}(25,90)\\ \;\;\;\;\;\;\;\;\;\;\;\;\;\;\;\;\;\;+0.002\times \text{I}\text{D}\text{\_}\text{G}\text{l}\text{o}\text{b}\text{a}\text{l}\text{\_}\text{M}\text{a}\text{x} \end{array}$$ (2)

$$\begin{array}{l} \text{T}\text{h}\text{e}\;\text{p}\text{a}\text{t}\text{i}\text{e}\text{n}\text{t}-\text{b}\text{a}\text{s}\text{e}\text{d}\;\text{r}\text{i}\text{s}\text{k}\;\text{s}\text{c}\text{o}\text{r}\text{e}\;\text{u}\text{s}\text{i}\text{n}\text{g}\;\text{c}\text{l}\text{i}\text{n}\text{i}\text{c}\text{a}\text{l}\;\text{f}\text{a}\text{c}\text{t}\text{o}\text{r}\text{s}\;\text{o}\text{n}\text{l}\text{y}\\ \;\;\;\;\;\;\;\;\;\;\;\;\;\;\;\;=\;-15.680+2.833\times \text{l}\text{e}\text{s}\text{i}\text{o}\text{n}\;\text{n}\text{u}\text{m}\text{b}\text{e}\text{r}+0.104\\ \;\;\;\;\;\;\;\;\;\;\;\;\;\;\;\;\times \text{l}\text{a}\text{c}\text{t}\text{a}\text{t}\text{e}\;\text{d}\text{e}\text{h}\text{y}\text{d}\text{r}\text{o}\text{g}\text{e}\text{n}\text{a}\text{s}\text{e}-1.674\\ \;\;\;\;\;\;\;\;\;\;\;\;\;\;\;\;\times \text{c}\text{r}\text{e}\text{a}\text{t}\text{i}\text{n}\text{e}\;\text{k}\text{i}\text{n}\text{a}\text{s}\text{e}\;\text{i}\text{s}\text{o}\text{e}\text{n}\text{z}\text{y}\text{m}\text{e}\text{s} \end{array}$$ (3)

$$\begin{array}{l} \text{T}\text{h}\text{e}\;\text{p}\text{a}\text{t}\text{i}\text{e}\text{n}\text{t}-\text{b}\text{a}\text{s}\text{e}\text{d}\;\text{r}\text{i}\text{s}\text{k}\;\text{s}\text{c}\text{o}\text{r}\text{e}\;\text{c}\text{o}\text{m}\text{b}\text{i}\text{n}\text{i}\text{n}\text{g}\;\text{r}\text{a}\text{d}\text{i}\text{o}\text{m}\text{i}\text{c}\text{s}\\ \;\;\;\;\;\;\;\;\;\;\;\;\;\;\;\;\;\;\;\text{a}\text{n}\text{d}\;\text{c}\text{l}\text{i}\text{n}\text{i}\text{c}\text{a}\text{l}\;\text{f}\text{e}\text{a}\text{t}\text{u}\text{r}\text{e}\text{s}\\ \;\;\;\;\;\;\;\;\;\;\;\;\;\;\;\;=-114.053+9.911\times \text{l}\text{e}\text{s}\text{i}\text{o}\text{n}\;\text{n}\text{u}\text{m}\text{b}\text{e}\text{r}+122.045\\ \;\;\;\;\;\;\;\;\;\;\;\;\;\;\;\;\times \text{G}\text{L}\text{R}\text{L}\text{M}\text{\_}\text{L}\text{R}\text{L}\text{G}\text{E}\text{\_}(25,90)+0.0196\\ \;\;\;\;\;\;\;\;\;\;\;\;\;\;\;\;\times \text{I}\text{D}\text{\_}\text{G}\text{l}\text{o}\text{b}\text{a}\text{l}\text{\_}\text{M}\text{a}\text{x}+0.334\;\\ \;\;\;\;\;\;\;\;\;\;\;\;\;\;\;\;\times \text{l}\text{a}\text{c}\text{t}\text{a}\text{t}\text{e}\;\text{d}\text{e}\text{h}\text{y}\text{d}\text{r}\text{o}\text{g}\text{e}\text{n}\text{a}\text{s}\text{e}-7.593\\ \;\;\;\;\;\;\;\;\;\;\;\;\;\;\;\;\times \text{c}\text{r}\text{e}\text{a}\text{t}\text{i}\text{n}\text{e}\;\text{k}\text{i}\text{n}\text{a}\text{s}\text{e}\;\text{i}\text{s}\text{o}\text{e}\text{n}\text{z}\text{y}\text{m}\text{e}\text{s} \end{array}$$ (4)

where GLRLM_LRLGE_(25, 90) represents the radiomic feature, long-run, low gray-level emphasis, which describes the distribution of the long homogeneous runs with low gray-levels within the image. The numbers in the brackets represent the parameters used to calculate that particular radiomic feature. ID_Global_max represents the radiomic feature intensity direct global max, which describes that the binary mask was preprocessed for the features derived directly from the image intensity. A detailed description of the parameters used is shown in Figure 2.

In contrast, the developed patient-based random forest models demonstrate comparable AUC values, precision, recall, specificity, F1, and accuracy as compared to the patient-based risk scores in the validation set (Table 3). The results of the decision curve analysis (DCA) to evaluate the clinical utility of the risk scores and the random forest models built in this study are shown in Figure 3E, 3F. The risk scores show a comparable clinical utility as compared to the random forest models.

### Lesion-wise COVID-19 risk score with radiomic features only

**Figure 4:**
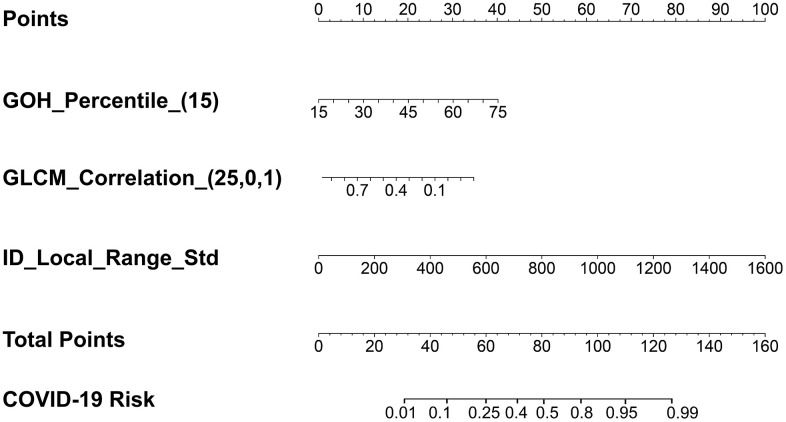
**The lesion-based risk score using three radiomic features only.** GOH_Percentile_(15) represents the radiomic feature gradient orient histogram, which describes the percentiles of the occurrence probability values in the histogram of the image. The numbers in the brackets represent the parameters used to calculate that particular radiomic feature. The parameter of 15 in GOH_Percentile represents the histogram percentile. GLCM_Correlation_(25,0,1) represents the radiomic feature gray-level co-occurrence matrix with statistical measurement of correlation between a pixel and its neighbor over the whole image, which describes that the gray-level co-occurrence matrix was computed from the image inside the binary mask in 2.5D with the direction of the angle of intensity pair at 0 degrees and the distance between the intensity pairs at 1. ID-Local_Range_Std represents the intensity direct in the neighborhood region, which describes the standard deviation among all the voxels.

**Figure 5:**
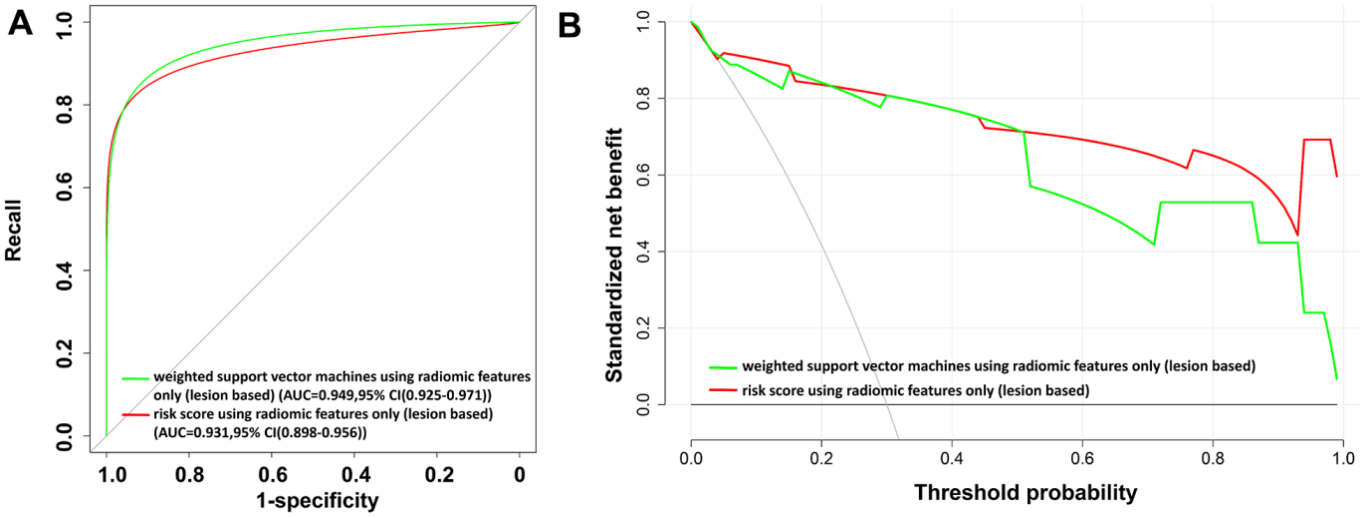
**The receiver operating characteristic (ROC) curves and the decision curve analysis (DCA) for the lesion-based risk score and weighted support vector machine model using radiomic features alone.** (**A**) ROC curve. (**B**) DCA analysis. In (**B**), the *x*-axis of the decision curve is the threshold of the predicted probability using the risk score to classify COVID-19 and non-COVID-19 patients. The *y*-axis shows the clinical decision net benefit for patients based on the classification result in this threshold. The decision curves of the treat-all scheme (the monotonically decreasing dash-line curve in the figure) and the treat-none scheme (the line when *x* equals zero) are used as references in the DCA. In this study, the treat-all scheme assumes that all patients had COVID-19; the treat-none scheme assumes that none of the patients had COVID-19. Abbreviations: AUC, area under the curve; 95% CI, 95% confidence interval.

To characterize different infectious lesions within the same patient, a lesion-based risk score using three radiomic features alone was also constructed (Figure 4 and Equation (5)). The utility of the risk score achieved an AUC value of 0.931 (95% CI: 0.898–0.956) (Table 4 and Figure 5A).

**Table 4:** The diagnosis performance using the lesion-based risk score and weighted support vector machine.

**Signature**	**AUC**	**Precision**	**Recall**	**Specificity**	**F1 score**	**Accuracy**	**Delong test for AUC**
COVID-19 risk score	0.931 (0.898–0.956)	0.976 (0.944–0.996)	0.891 (0.831–0.927)	0.921 (0.872–0.965)	0.927 (0.901–0.966)	0.902 (0.834–0.981)	Z = 4.371, P < 0.000
Weighted support vector machine	0.949 (0.925–0.971)	0.969 (0.923–0.981)	0.904 (0.824–0.936)	0.942 (0.899–0.966)	0.935 (0.876–0.964)	0.987 (0.886–0.995)	

The values in the brackets represent the 95% confidence interval (95% CI).

Abbreviations: AUC, area under the curve.

$$\begin{array}{l} \text{T}\text{h}\text{e}\;\text{l}\text{e}\text{s}\text{i}\text{o}\text{n}\\ -\text{b}\text{a}\text{s}\text{e}\text{d}\;\text{r}\text{i}\text{s}\text{k}\;\text{s}\text{c}\text{o}\text{r}\text{e}\;\text{u}\text{s}\text{i}\text{n}\text{g}\;\text{r}\text{a}\text{d}\text{i}\text{o}\text{m}\text{i}\text{c}\text{s}\;\text{f}\text{e}\text{a}\text{t}\text{u}\text{r}\text{e}\text{s}\;\text{a}\text{l}\text{o}\text{n}\text{e}\\ =\;-55.389-6.769\times \text{G}\text{L}\text{C}\text{M}\_\text{C}\text{o}\text{r}\text{r}\text{e}\text{l}\text{a}\text{t}\text{i}\text{o}\text{n}\_(25,0,1)\\ +0.33\times \text{I}\text{D}\text{\_}\text{L}\text{o}\text{c}\text{a}\text{l}\text{\_}\text{R}\text{a}\text{n}\text{g}\text{e}\text{\_}\text{S}\text{t}\text{d}+0.136\\ \times \text{G}\text{O}\text{H}\text{\_}\text{P}\text{e}\text{r}\text{c}\text{e}\text{n}\text{t}\text{i}\text{l}\text{e}\_(15) \end{array}$$ (5)

where GLRLM_Correlation_(25,0,1) represents the radiomic feature gray-level co-occurrence matrix with statistical measurement of correlation between a pixel and its neighbor over the whole image. The numbers in the brackets represent the parameters used to calculate that particular radiomic feature. ID_Local_Range_Std represents the radiomic feature of intensity direct in the neighborhood region, which describes the standard deviation among all the voxels. GOH_Percentile_(15) represents the radiomic feature gradient orient histogram, which describes the percentiles of the occurrence probability values in the histogram of the image. A detailed description of the parameters used is shown in Figure 4.

In contrast, the lesion-based weighted support vector machine (WSVM) model using the radiomic features only demonstrates a comparable AUC value, precision, recall, specificity, F1, and accuracy as compared to the lesion-based risk score (Table 4). The results of the DCA analysis to evaluate the clinical utility of the risk score and the WSVM model using radiomic features only are shown in Figure 5B.

## DISCUSSION

During the incubation period of SARS-CoV-2, before the onset of clinical symptoms confirmed by positive nucleic acid detection, about 96% of patients would have non-specific CT imaging changes similar to other viral pneumonias in the lungs, i.e., GGO, patchy consolidation, and sub-solidification [4, 9, 11, 12]. In this study, the performance of the radiomics-based risk scores was compared to that of human diagnosis in differentiating COVID-19 from viral pneumonia. We demonstrated that both the patient-based risk score using radiomic features only and the lesion-based risk score using radiomic features only have significantly better classification abilities than the human diagnosis at the patient- and lesion-wise levels. This can partially be attributed because without the aid of other clinical information, radiologists might achieve a relatively low sensitivity and specificity in differentiating COVID-19 from viral pneumonias based only on the chest CT manifestations.

The risk score could provide a quantitative measure to appropriately adjust the cut-off value based on desired levels of recall and specificity to reduce the adverse consequences of false negatives in the differentiation of COVID-19. In addition, with the quantitative measurements used, it might be useful to longitudinally monitor disease progress over time or recurrence in the recovered COVID-19 patients using delta radiomics methods, although this possibility is still under investigation.

In the patient-based COVID-19 risk scores, three clinical variables, i.e., lesion number, LDH activity, and CK-MB activity, show discriminative abilities for COVID-19 detection. Notably, the imaging pattern showing a multifocal appearance with a lesion number larger than 3–5 could be used as a rapid cut-off in the case of a strong suspicion of SARS-CoV-2 infection. Meanwhile, LDH serves as an inflammatory predictor in many pulmonary diseases, such as obstructive disease, microbial pulmonary disease, and interstitial pulmonary disease [13, 14]. A recent study showed that refractory COVID-19 patients had increased blood LDH and CRP levels. Moreover, another study demonstrated that COVID-19 patients treated in the ICU had higher levels of LDH and CRP than those not treated in the ICU [15]. These observations suggest that LDH levels might reflect the acute severe systemic inflammatory response involved in cell-mediated immunity and cytokine storms caused by SARS-CoV-2 infection, which is a distinguishable biochemical parameter for inflammation in the risk score.

Furthermore, a previous study suggested that the increases in LDH and CK-MB levels were correlated with SARS-CoV-2 mRNA levels in RT-PCR positive patients [16]. As such, all three clinical variables in the risk scores might emphasize the underlying biological mechanism(s) related to COVID-19. The immunological mechanism of SARS-CoV-2 infection still requires further investigation.

In the patient-based COVID-19 risk scores, two radiomic features, GLRLM_LRLGE_(25,90) and ID_Global_max, were selected to build the risk score with significantly strong discriminative abilities for COVID-19 detection (i.e., the features with *P* < 0.001 in the multivariable logistic regression). GLRLM_LRLGE had a higher weight (larger coefficient) in the risk score compared to other radiomics and clinical features. GLRLM_LRLGE analyzes the spatial information within chest CT image runs in the upper right quadrant of the GLRLM with long run lengths and low gray-levels. The longer runs with different gray-level intensities are closely linked with coarse texture and regional heterogeneity as compared to fine texture [17]. Therefore, GLRLM_LRLGE might be associated with the coarseness of COVID-19 [18]. Consequently, a higher GLRLM_LRLGE, i.e., a coarser texture on chest CT images, may be associated with a higher risk of occurrence of COVID-19 [19].

Interestingly, three different radiomic features, GOH_Percentile_(15), GLCM_Correlation_(25,0,1), and ID_Local_Range_Std, were identified in our lesion-based analysis. In particular, previous studies suggested that a GLCM_correlation value might be inversely related with the levels of vascular endothelial growth factor (VEGF), which controls critical physiological functions in the lung [20–22]. For example, a decrease in VEGF expression is believed to be associated with acute lung injury and alkaloid monocrotaline-pulmonary hypertension [23], which is one of the most common comorbidities in COVID-19 [24]. However, the relationship between radiomic features and the phenotypes linked to COVID-19 is not well understood at present.

There are different processing methods for patient-based analysis. Some studies selected the largest lesion and/or the most metabolically active lesion as a representative lesion for that patient based on a method reported previously [25, 26]. However, the large heterogeneous lesions (often necrotic and/or with multiple uptake peaks) may underestimate image texture measurements [27]. Nevertheless, all the lesions could be used for radiomics analysis, which enriches the analysis through the use of the information derived from all lesions. However, the method of averaging the radiomic values of all lesions as the characteristic value for one particular radiomic feature could dilute the feature value of large lesions by other small lesions. As such, in this study, a weighted power mean method was used in the patient-based analysis to emphasize that the lesions with relatively large volume represent the main characteristics of the biological behavior and characteristics of the disease type, while still retaining the other small lesions representing a certain kind of disease progression. In contrast, the lesion-based analysis allowed us to examine each individual lesion with a consideration of different infectious lesions within the same patient.

There could be certain bias introduced in the boundary and volume contoured in the manual delineating process by different radiologists, which could certainly affect the radiomics values calculated. However, this kind of inter-observer variation mainly influences the shape-related radiomic features. It has relatively limited influence on the features of GLRLM_LRLGE_(25, 90), ID_Global_Max, GOH_Percentile_(15), GLCM_Correlation_(25,0,1), and ID_Local_range_std selected in this study. A previous study conducted by eight research centers in the United States and one medical imaging center in Canada suggested that the segmentation mainly affects the global shape descriptors features, but has relatively little effect on the texture and intensity features of the entire three-dimensional volume [28]. Also, GLRLM_LRLGE_(25, 90), ID_Global_Max GOH_Percentile_(15), GLCM_Correlation_(25,0,1), and ID_Local_range_std are five important features in the texture and intensity features category. A verification study is described in Supplementary Appendix 3.

As a retrospective study, this study has several limitations. First, the patient cohort in this study is relatively small. The use of digital biopsy technologies with promising retrospective radiomics analyses must still be further evaluated in prospective clinical trials, thus facilitating a better personalized patient management. Second, all patients are from Zhejiang Province, China, and might not fully represent the spectrum of COVID-19 phenotypes. The relationships between radiomic features and their underlying immune interactions and biological mechanism(s) directing COVID-19 progression at the early stage of SARS-CoV-2 infection also remain to be explored. Third, although the radiomic features and clinical variables associated with disease progression were not evaluated in this study, the findings of this research may still provide useful insights for future studies to identify the underlying mechanism(s) and relevant radiomic features for disease severity, prognosis, and patient outcome of SARS-CoV-2 infection.

## CONCLUSIONS

The point-of-care COVID-19 risk scores could be an easy-to-use tool to quantitatively differentiate COVID-19 from other viral pneumonias. The risk scores using chest CT radiomic features and/or clinical characteristics could better characterize the SARS-CoV-2 infection landscape, which still significantly overlaps with other virus-induced pneumonias in visual inspection of CT manifestations. The risk scores developed could potentially afford a clinically translatable means to improve the diagnostic confidence using chest CT for COVID-19 detection in the future.

## MATERIALS AND METHODS

### Patients

This study was approved by the Hangzhou Xixi Hospital Institutional Review Board. As this is a retrospective study, the need for written informed consent from patients was waived. A total of 193 patients confirmed with COVID-19 or other types of viral pneumonia were enrolled in this study. Eight patients with negative chest CT imaging result were excluded. A total of 108 patients with COVID-19 confirmed by RT-PCR between December 2019 and March 2020 in the Hangzhou Xixi Hospital were retrospectively included into this study. Another group of 77 patients with influenza virus-induced, adenovirus-induced, syncytial virus-induced, and cytomegalovirus-induced pneumonias from Hangzhou First People’s Hospital (19 cases) and Hangzhou Xixi Hospital (58 cases) were used as controls.

**Figure 6:**
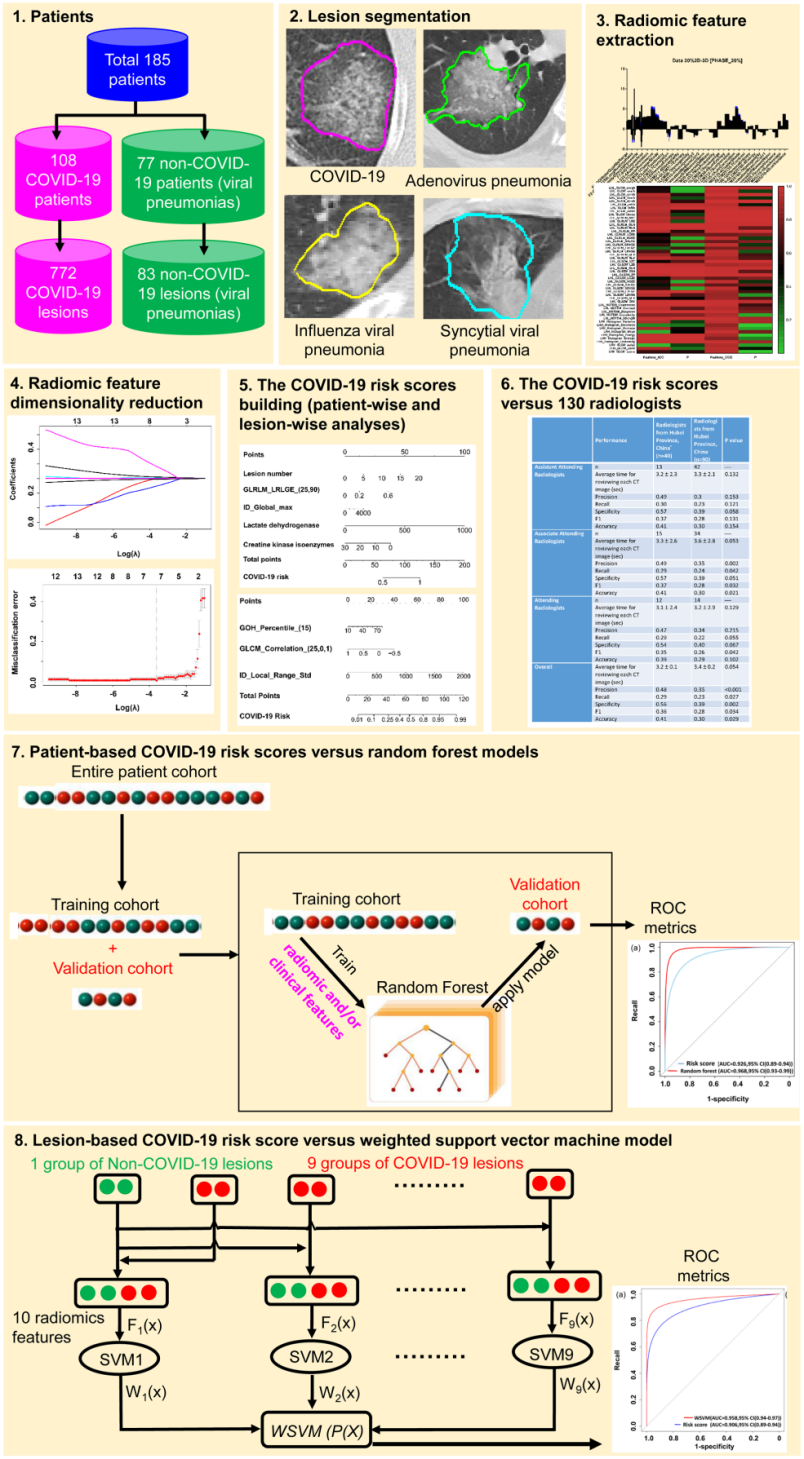
**The workflow for the development and validation of COVID-19 risk scores.**

The patients’ electronic medical data were retrieved from the Hospital Information System (HIS). The high-resolution CT images were retrieved from the picture archiving and communication system of the hospitals. The patients’ RT-PCR results were retrieved from the electronic medical records in the HIS. The patients with negative chest CT results or lacking both chest CT and RT-PCR examinations were excluded from this study. Figure 6 summarizes the study workflow and methods.

Baseline clinical data, including patient’s age, gender, lesion number, and five biochemical indicators recommended in the “Handbook of COVID-19 Prevention and Treatment,’’ [29] including white blood cell count (WBC), C-reactive protein (CRP) levels, creatine kinase isoenzyme (CK-MB) activity, lactate dehydrogenase (LDH) activities, and plasma D-dimer (DD) levels, were collected by reviewing the medical records and data of serial CT imaging, including baseline, mid-treatment, and post-treatment CT scans, were also recorded to monitor the disease progression. The patients’ daily basic status, daily examination results, and complications were also analyzed to check how the disease progressed. Based on the RT-PCR results for COVID-19 confirmation, the enrolled patients were divided into two groups, i.e., the COVID-19 group and the non-COVID-19 viral pneumonia group.

### CT image acquisition

All patients included underwent chest CT imaging using a two multi-detector row CT system (GE Revolution Evo CT, Chicago, USA; Siemens SOMATOM Emotion 16, Erlangen, Germany). The acquisition parameters were as follows: 120/130 kV, 100/10–240 mA, 0.35- or 0.8-second rotation time, a layer spacing of 5 mm, an acquisition layer thickness of 5 mm, high-resolution reconstruction with a lung window layer thickness of 1.25/1.50 mm, a detector collimation of 16×0.5 mm or 64×0.625 mm, a field of view of 350×350 mm, and an image matrix of 512×512. The CT scans before onset of symptoms or CT scans done ≤1 week after symptom onset were used as baseline. The baseline CTs of highly suspected patients were used in this study. GGO and/or consolidation are the main manifestations in the CT images at this early stage. The other CT imaging patterns included linear opacity, mixed type and interstitial change patterns including septal thickening and fine reticular opacity, and other features including vascular thickening, crazy paving pattern, pleural thickening, and pleural effusion.

### Human diagnosis of COVID-19 using a human supervised learning fashion

To compare the classification performance between the COVID-19 risk scores developed and radiologists, 147 radiologists were invited to differentiate COVID-19 from the virus-induced pneumonias based on the CT manifestations only. The diagnosis was performed using a human supervised learning fashion.

A total of five COVID-19 CT images and five influenza virus-induced, adenovirus-induced, syncytial virus-induced, and cytomegalovirus-induced pneumonia images were randomly drawn from the total patient cohort to form a learning sample set. These learning sample images along with the CT manifestations described in the China Clinical Consensus on Radiological Diagnosis on COVID-19 [29] were used to train 130 radiologists in China with thoracic CT diagnosis experiences ranging from assistant attending radiologists or associate attending radiologists to attending radiologists using a human supervised learning fashion. The radiologists were then given the remaining 176 CT images without the clinical and follow-up information provided. Based on the CT manifestations they learned from the 10 image samples provided, the radiologists diagnosed whether these 176 CT images were COVID-19 or influenza virus-/adenovirus-/syncytial virus-/cytomegalovirus-induced pneumonias. The accuracy and the average time for diagnosis per CT image were used for statistical analysis. To rule out the random diagnosis, two equal CT images were mixed within the 176 CT images, and if the radiologist’s answers were not consistent for these two images, his or her answers were excluded from the statistical analysis. A total of 130 radiologists’ diagnoses were eligible for statistical analysis (40 radiologists from Hubei Province, the epicenter of the COVIA-19 outbreak in China, and 90 radiologists from outside of Hubei Province).

### Radiomic feature extraction

Before extracting all chest CT radiomic features, 3D adaptive histogram equalization enhancement (AHEE-3D) and edge preserve smooth 3D (EPS-3D) methods were used to remove random noise in the images. The lesions of pneumonias on CT images were reviewed and manually delineated by two experienced attending radiologists who were blind to the clinical and follow-up information. The final contour for each lesion was agreed upon by both radiologists. The patient-based and lesion-based analyses were performed. The lesion region of interest (ROI) was segmented on the CT image as the only input for radiomics analysis of pneumonia.

A total of 1766 radiomic features were extracted from each ROI delineated using the image biomarker explorer (IBEX) public platform developed by the University of Texas MD Anderson Cancer Center for feature extraction and classification of radiomic features [30, 31]. The radiomic features extracted include seven categories: shape, intensity direct, intensity histogram, gray-level co-occurrence matrix (2.5D and 3D), neighbor intensity difference (2.5D and 3D), gray-level run length matrix (2.5D), and intensity histogram Gauss fit.

It is believed that some of the radiomic features are sensitive to each step of the data processing procedure, including image acquisition settings, image reconstruction algorithm, and the digital image preprocessing procedure, so that the repeatability and reproducibility of the extraction of these radiomic features are easily compromised [32]. To account for the potential impact of the accuracy of radiomic feature extraction, the radiomic feature extraction procedure was repeated twice and Lin’s concordance correlation coefficient (CCC) tests were performed to assess the feature reproducibility in repeated feature extraction [33, 34]. Only the 1237 radiomic features showing high CCC values (CCC > 0.99) were used. With 1237 radiomic features selected, 510 features with null value were eliminated and the remaining 727 radiomic features plus 9 clinical variables (lesion number, age, gender, WBC, LC, CRP level, LDH activity, CK-MB activity, and plasma DD level) were used for further analysis.

### Patient-based risk scores

Of the patient data, 25% was randomly selected as an independent validation set (*n* = 46) and the remaining 75% of the patient data were used for the training set (*n* = 139). The ratio of COVID-19 to non-COVID-19 patients was about 1.41:1 in the training and validation sets (in the training set, COVID-19:non-COVID-19 = 81:58 patients; in the validation set, COVID-19:non-COVID-19 = 27:19 patients).

For our patient-based analysis, the same features extracted from multiple lesions within one single patient were combined using a weighted power mean method [35]. Briefly, all lesions of the patient were delineated and the radiomic features were extracted. A weighting calculation was performed to combine the same feature from different lesions within the same patient as described in the following equations (Equation 6):

$$\begin{array}{l} \text{F}(\text{j})=\sum_{\text{i}=1}^{\text{n}}\frac{{\text{V}}_{\text{i}}}{{\text{V}}_{\text{T}}}\cdot {\text{f}}_{\text{j}}(\text{i})\\ {\text{V}}_{\text{T}}=\sum_{\text{i}=1}^{\text{n}}{\text{V}}_{\text{i}}, \end{array}$$ (6)

where *F*(*j*) represents the value of the *j*-th radiomic feature of the patient, *i* represents the *i*-th lesion of the patient, *V*(*i*) represents the volume of the *i*-th lesion, *n* represents the number of lung lesions in the patient, *V*_T_ represents the total volume of all lesions in the patient, and *f*_j_(*i*) represents the value of feature *j* in the *i*-th lesion. The weight assigned was based on the volume of the lesion. The larger the lesion volume, the greater the weight value of the features extracted from that lesion. Thus the contribution of the features extracted from this lesion to the patient’s radiomic feature was also greater.

Owing to the imbalanced sample distribution between COVID-19 and non-COVID-19 patients (the number of non-COVID-19 patient is lower than the number of COVID-19 patients), synthetic minority over-sampling technology [36–40] was used to generate synthetic non-COVID-19 patient samples in the training set so that a synthetically class-balanced training set could be achieved prior to training the models in this study. Briefly, for each minority sample “a” in the non-COVID-19 patient group, the synthesis strategy was applied to randomly select a minority sample “b” from its nearest neighbors. And then one point was randomly selected as the newly synthesized non-COVID-19 patient sample on the line between “a” and “b,” so that the ratio of COVID-19 and non-COVID-19 patients in the training set was close to 1:1.

**Figure 7:**
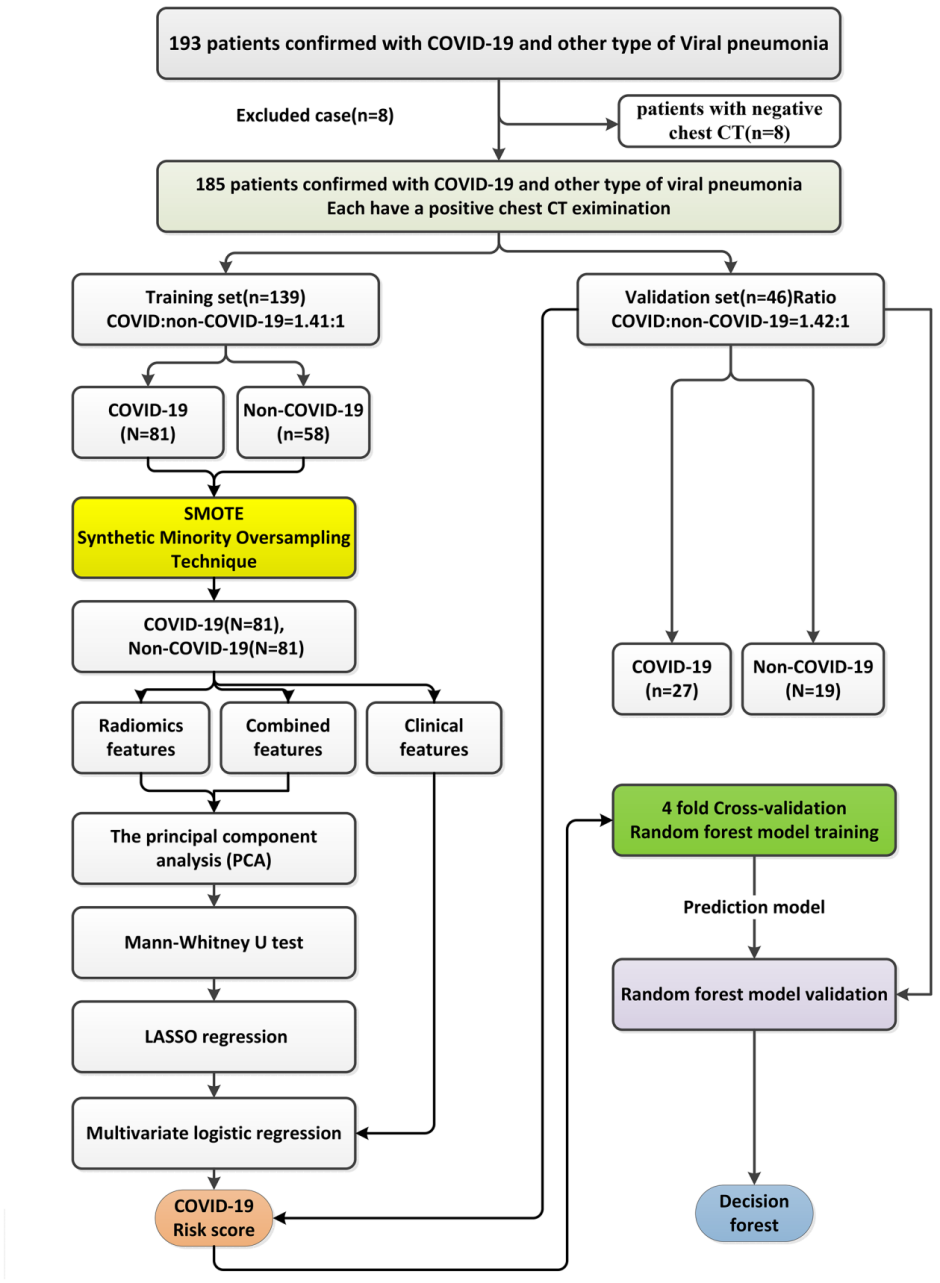
**The workflow of the construction of the patient-based risk scores using radiomic features only, the risk score using clinical factors only, and the risk score combining radiomic features and clinical variables using a multivariate logistic regression method and a random forest model.**

Three signatures, including a risk score using radiomic features only, a risk score using clinical factors only, and a risk score combining radiomic features and clinical variables, were built in this study (Figure 7). For the construction of the risk score using radiomic features only and the risk score combining radiomic features and clinical variables, principal component analysis (PCA), the Mann–Whitney U test, and least absolute shrinkage and selection operator (LASSO) regression with a four fold cross-validation method and a 100 times iterative selection process were successively applied to eliminate redundant features and irrelevant variables to establish the COVID-19 risk scores. A multivariate logistic regression method was used to build these two risk scores. For the construction of the risk score using clinical factors only, because the number of clinical factors is much lower than the number of radiomic features, a multivariate logistic regression method was directly applied to build the clinical signature. The model performance of the three risk scores was evaluated in the training and independent validation sets.

For the construction of the risk score using radiomic features only and the risk score combining radiomic features and clinical factors, PCA was used to reduce the feature dimensionality and select the radiomic features and radiomic features plus clinical variables that accounted for 90% of the significant feature subset variability to increase the discriminative ability. After PCA analysis, the feature dimensionality was reduced from 727 radiomic features to 32 features for the risk score using radiomic features only (Supplementary Table 1), and from 727 radiomic features plus 9 clinical variables to 26 features for the risk score combining radiomic features and clinical factors (20 radiomic features plus 6 clinical variables including lesion number, gender, WBC, CRP level, LDH activity, CK-MB activity, and plasma DD level) (Supplementary Table 2).

The Mann–Whitney U test was used to further explore the potential association of the features/variables selected from PCA with COVID-19 and further reduce the feature dimensions. For the risk score using radiomic features only (Supplementary Table 3), the feature dimensions were reduced from 32 to 20 radiomic features. For the risk score combining radiomic features and clinical factors, the feature dimensions were reduced from 26 to 17 features (11 radiomic features plus 6 clinical variables including lesion number, gender, WBC, CRP level, LDH activity, and CK-MB activity) (Supplementary Table 4).

To select the most suitable features for classification of COVID-19, LASSO regression with a four fold cross-validation method and a 100 times iterative selection process was used to continually choose non-redundant and the most robust radiomic features and radiomic features and clinical variables, respectively [41, 42]. The coefficient of each variable was controlled by the parameter λ in the LASSO method and only the features with non-zero coefficients were selected. The misclassification error was calculated to minimize the binary classification error and maintain a balance of optimal classification performance and the optimal number of radiomic features needed for binary classification (COVID-19 vs. non-COVID-19).

As such, for the risk score using radiomic features only, only those 16 features with non-zero coefficients were selected via the LASSO process (Supplementary Table 5 and Supplementary Figure 1). For the risk score combining radiomic features and clinical variables, only those five features with non-zero coefficients were selected via the LASSO process (two radiomic features, GLRLM_LRLGE_(25,90) and ID_Global_Max, plus three clinical variables, lesion number, LDH activity, and CK-MB activity) (Supplementary Table 6 and Supplementary Figure 2).

After the feature dimensionality was reduced, a multivariable logistic regression analysis was employed and only the features with *P* < 0.001 in this process were selected to build the COVID-19 risk scores. For the risk score using radiomic features only, two radiomic features, GLRLM_LRLGE_(25,90) and ID_Global_Max, were finally preserved (Supplementary Table 7). For the risk score combining radiomic features and clinical variables, seven features were further reduced to five features (two radiomic features, GLRLM_LRLGE_(25,90) and ID_Global_Max, plus three clinical variables, lesion number, LDH activity, and CK-MB activity) (Supplementary Table 8). The COVID-19 risk scores using radiomic features only and using radiomics and clinical variables were built as the final classifiers by summing these features multiplied with their respective coefficients.

The COVID-19 risk scores developed were also represented by nomograms. The threshold of using the risk score using radiomic features only to classify COVID-19 is 0.2. The threshold of using the risk score with combined radiomic and clinical variables to classify COVID-19 is 0.5. DCA was applied to evaluate the clinical decision utility of the COVID-19 risk scores developed [34, 35]. The definition of net benefit in the DCA is described in Supplementary Appendix 1.

For the risk score using clinical factors only, the multivariable logistic regression analysis was directly employed and only the variables with *P* < 0.001 in this process were selected to build the COVID-19 risk score, including lesion number, LDH activity, and CK-MB activity (Supplementary Table 9). The COVID-19 risk score using clinical factors only was also represented by a nomogram. The threshold using a nomogram to classify COVID-19 is 0.5.

### Patient-based random forest models

As a comparison to the patient-based COVID-19 risk scores developed, three random forest classifiers using radiomic features only, clinical factors only, and a combination of radiomic features and clinical factors were also constructed using grid search with fourfold cross-validation with the following parameters: the number of trees in the forest (ntree) = 500 and the maximum depth of the tree (mtry) = 3.

### Lesion-wise COVID-19 risk score with radiomic features only

A lesion-based COVID-19 risk score using radiomic features alone was also built so that potentially different infectious lesions could be characterized individually. In total, 772 COVID-19 lesions were extracted from COVID-19 patients and 83 non-COVID-19 lesions were extracted from related viral pneumonia patients in this study.

The feature dimensionality reduction was conducted to select the optimal radiomic features. Briefly, a total of 1766 radiomic features were extracted from each lesion individually. After eliminating the radiomic features with null values and employing PCA, 32 radiomic features were used for the Mann–Whitney U test (Supplementary Table 10). The Mann–Whitney U test further reduced the feature dimensions from 32 to 20 features (Supplementary Table 11). The LASSO regression further selected the 10 non-redundant and most robust radiomic features (Supplementary Table 12 and Supplementary Figure 3).

After the feature dimensionality was reduced, the multivariable logistic regression analysis was employed to choose the radiomic features with *P* < 0.001 to build the lesion-based COVID-19 risk score with radiomic features alone so that 10 features were further reduced to 3 features: GLCM_Correlation_(25,0,1), ID_Local_Range_Std, and GOH_Percentile_(15) (Supplementary Table 13). The lesion-based COVID-19 risk score based on three features only was also represented by a nomogram. The threshold of using the risk score to classify COVID-19 is 0.5. DCA was also employed to evaluate the clinical decision utility of the nomogram developed [43, 44].

### Lesion-based weighted support vector machine analysis

As a comparison to the lesion-based COVID-19 risk score using radiomic features alone, a lesion-based WSVM analysis was also conducted using the 10 radiomic features (Supplementary Table 12) selected by the LASSO. The data distribution between the COVID-19 and non-COVID-19 lesions (approximately 9.3:1) was extremely imbalanced. To ensure the models with predictive power were equally balanced between COVID-19 and non-COVID-19, a previously described strategy was used to adjust the distribution imbalance between COVID-19 and non-COVID-19 lesions and construct the WSVM [45].

Briefly, the strategy is to separate the major class (i.e., the COVID-19 lesion group in this study) into small subset groups size-comparable to the minor class (i.e., the non-COVID viral pneumonia lesion group in this study) to achieve a balanced distribution between the major class and the minor class; the COVID-19 lesion groups was randomly decomposed into nine partitions, and all the non-COVID-19 lesions were combined with each partition of COVID-19 lesions to form an individual subset so that the ratio of the COVID-19 and non-COVID-19 lesions was nearly 1:1 in each individual subset. In each individual subset, the total lesions were randomly separated into the training set (70%) and the validation set (30%).

The support vector machine (SVM) was trained independently with 10 radiomic features selected by the LASSO process within the training set of each subset. The weight for the SVM was determined via the recall value of the prediction using the validation set to reduce the false negative rate. In each individual subset, the SVM generated was validated with the validation set of each subset (i.e., the balanced data of each subset) as well as the validation set of the entire data to evaluate the classification performances (Supplementary Table 14 and Supplementary Figure 4).

Finally, all constituent SVMs were combined by summing constituent SVMs multiplied by weights determined, divided by the sum of the weights. The classical (metric) multidimensional scaling matrix (CMDScale) was used to demonstrate the correlation of features and COVID-19 for each constituent SVM.

### Performance evaluation and statistical analysis

The AUC between the risk scores and the random forest models and the WSVM model was compared using the Delong test. Six metrics, including precision, recall (sensitivity), specificity, F1, accuracy, and AUC, were calculated from the receiver operating characteristic (ROC) curve with the model output.

The classification performances of COVID-19 by the developed risk scores were assessed by ROC analysis. For numeric variables, mean and standard deviation were calculated and the differences between COVID-19 and non-COVID-19 patient groups were compared using rank-sum tests. A two-tailed *P* value less than 0.05 was regarded as statistically significant.

### Data sharing

The datasets analyzed in this study will be available from the corresponding author (Xiadong Li, email: lixiadong2019@outlook.com) at the time of publication. Per institutional policy, the datasets are designated limited access. Upon receiving access, the investigator may only use them for the purposes outlined in the request to the data provider, and redistribution of the data is prohibited.

## Supplementary Material

Appendices

Supplementary Figures

Supplementary Tables 1 to 14

Supplementary Table 15

Supplementary Table 16
