# Supplementary material for: A COVID-19 risk score combining chest CT radiomics and clinical characteristics to differentiate COVID-19 pneumonia from other viral pneumonias
Source: Aging (Albany NY). 2021 Mar 13;13(7):9186–224. doi: 10.18632/aging.202735 (PMC8064216; doi:10.18632/aging.202735)
Supplement: Appendices [file aging-13-202735-s001.pdf]

SUPPLEMENTARY APPENDIX 1

Definition of net benefit in the decision curve analysis

The net benefit [1, 2] was defined by the following equation:

$$\text{Net Benefit} = \text{TPR} * \omega - \frac{P_t}{1-P_t} * FPR * (1-\omega) \qquad (1)$$

P_t is the "threshold possibility" to stratify the patients into high-risk COVID-19 or low-risk non-COIVD-19 groups. Patients with a probability of having COVID-19 higher than P_t are high-risk patients. TPR is the true positive rate, defined as the proportion of high-risk patients in the patients having COVID-19. FPR is the false positive rate, defined as the proportion of high-risk patients in the patients having non-COVID-19. ω is the prevalence of having COVID-19, calculated by dividing the total patients number by the number of patients with COVID-19. In this study, the treat-all scheme assumes that all the patients were COVID-19; the treat-none scheme assumes that all the patients were non-COVID-19. In the condition of "treat none", no patient is classified as high risk, both the TPR and FPR are zero, so the Net Benefit is zero. In the condition of "treat all", all patients are classified as high risk COVID-19 (TPR=FPR=1), so the Net Benefit is calculated as

$$\begin{aligned}\text{Net Benefit}_{\textit{Treat Everyone}} &= \omega - \frac{P_t}{1-P_t} * (1-\omega) \\ &= \frac{1-\omega}{P_t - 1} + 1\end{aligned} \qquad (2)$$

, which is a monotonically decreasing dash-line curve in the figure.

SUPPLEMENTARY APPENDIX 2

The procedure of using the nomogram of the risk score combining radiomic features and clinical factors

This is the patient-based risk score integrating 2 radiomic features and 3 clinical variables. For example, a suspicious patient was found having the following radiomic features and clinical factors detected/calculated: lesions numbers = 5, GLRM_LRLGE_(25,90) = 0.3, ID_Global_Max = 2000, lactate dehydrogenase = 750 u/mg, creatine kinase isoenzymes = 10 ug/L. The values in the Points line in the 1st row corresponding to these radiomic features and clinical factors are 28, 58, 17, 32, 48. As such, the total point adding all the values in the Points line is 183 in the Total points line in the 7th row. So, the patient's risk of COVID-19 can be calculated from this nomogram with a risk score close to 0.95. Alternatively, all radiomic features and clinical factors detected/calculated can be plugged into the risk score equation to get the score value:

$$\begin{aligned}
&\text{The patient} - \text{based risk score combining radiomics} \\
&\quad \text{and clinical features} \\
&= -114.053 + 9.529 \times \text{lesion number} + 122.045 \\
&\quad \times \text{GLRLM_LRLGE_(25,90)} + 0.0196 \\
&\quad \times \text{ID_Global_Max} + 0.334 \\
&\quad \times \text{lactate dehydrogenase} - 7.593 \\
&\quad \times \text{creatine kinase isoenzymes} \\
&= -114.053 + 9.529 \times 5 + 122.045 \\
&\quad \times 0.3 + 0.0196 \times 2000 + 0.334 \times 750 \\
&\quad - 7.593 \times 10 = 183.9755
\end{aligned} \tag{3}$$

The score of 183.9755 is corresponding to 0.95 on the COVID-19 Risk line in the 8th row of the nomogram.

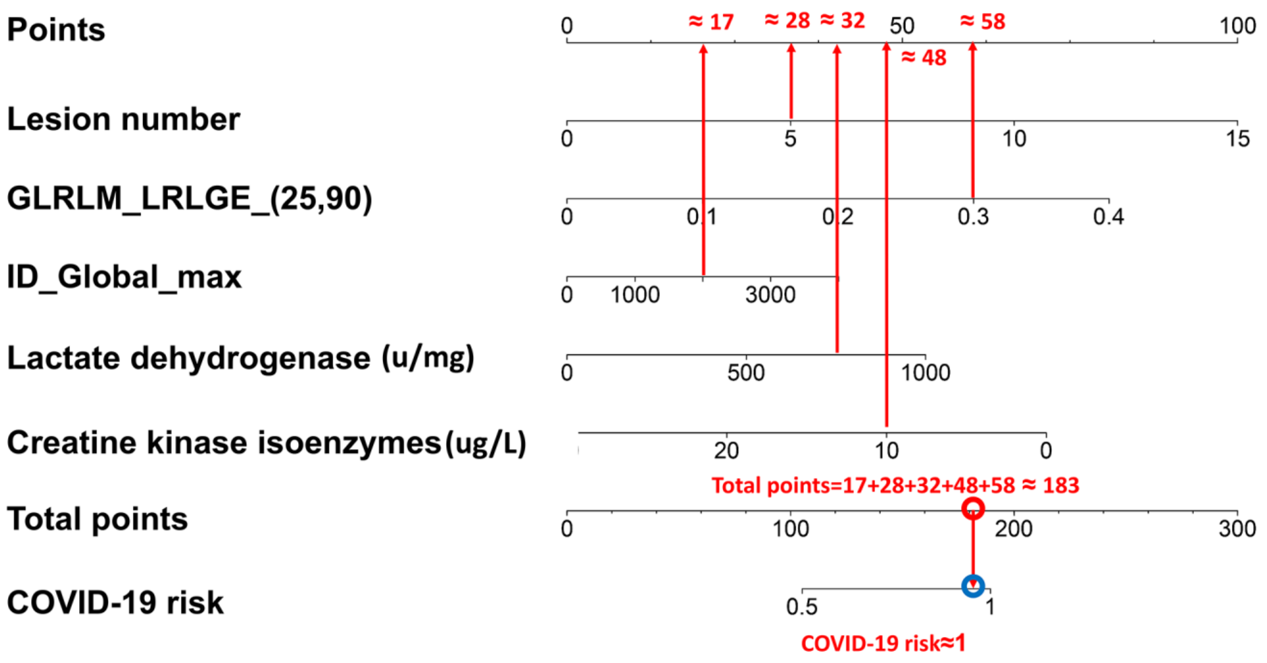

SUPPLEMENTARY APPENDIX 3

The influence of bias induced in the boundary and volume contoured in the manual delineating process on the radiomics values calculated

To further verify the repeatability of two features GLRLM_LRLGE_ (25, 90) and ID_Global_Max selected in the construction of patient-based risk scores in this study, a verification study was conducted. A CT data set from one COVID-19 patient was used for delineation by five different radiologists and the differences of volumes and surface areas caused by different delineations were calculated (Supplementary Figure 6). The 5 volumes-of-interest (VOIs) delineated were also used for the GLRLM_LRLGE_ (25, 90) and ID_Global_Max feature extraction and calculation. Three different tools (2 open sources ((image biomarker explorer (IBEX) [3] and Pyradiomics [4]) and 1 in-house Matlab codes) were used to extract and calculate the radiomic feature values of the GLRLM_LRLGE_ (25, 90) and ID_Global_Max. The patient-based COVID-19 risk score using radiomic features only was calculated using the radiomic features extracted from 5 VOIs contoured according to the formula developed in this study (Equation 5). The results are shown in Supplementary Table 15 as follows.

$$\text{The patient-based risk score using radiomic features only} = -3.785 + 19.563 \times \text{GLRLM\_LRLGE\_}(25,90) + 0.002 \times \text{ID\_Global\_Max}$$

As shown in Supplementary Table 15, the VOI delineation biases induced by different radiologists had a relatively small impact on the radiomic feature values of GLRLM_LRLGE_ (25, 90) and ID_Global_Max as well as the COVID-19 risk score values calculated. In addition, the feature extractions on the VOIs contoured using different tools had not significantly affected the calculation of COVID-19 risk score, which could be considered to be a good repeatability.

Furthermore, the same verification study was also repeated on three radiomic features identified in the lesion based analysis and the results were shown in Supplementary Table 16. Similarly, the VOI delineation biases induced by different radiologists had a relatively small impact on the radiomic feature values of GOH_Percentile_(15), GLCM_Correlation_(25,0,1) and ID_Local_range_std as well as the COVID-19 risk score values calculated. In addition, the feature extractions on the VOIs contoured using different tools had not significantly affected the calculation of COVID-19 risk score.

REFERENCES

1. Kerr KF, Brown MD, Zhu K, Janes H. Assessing the Clinical Impact of Risk Prediction Models With Decision Curves: Guidance for Correct Interpretation and Appropriate Use. J Clin Oncol. 2016; 34:2534–40. https://doi.org/10.1200/JCO.2015.65.5654 PMID:27247223
2. Vickers AJ, Van Calster B, Steyerberg EW. Net benefit approaches to the evaluation of prediction models, molecular markers, and diagnostic tests. BMJ. 2016; 352:i6. https://doi.org/10.1136/bmj.i6 PMID:26810254
3. Zhang L, Fried DV, Fave XJ, Hunter LA, Yang J, Court LE. IBEX: an open infrastructure software platform to facilitate collaborative work in radiomics. Med Phys. 2015; 42:1341–53. https://doi.org/10.1118/1.4908210 PMID:25735289
4. Kalpathy-Cramer J, Mamomov A, Zhao B, Lu L, Cherezov D, Napel S, Echegaray S, Rubin D, McNitt-Gray M, Lo P, Sieren JC, Uthoff J, Dilger SK, et al. Radiomics of Lung Nodules: A Multi-Institutional Study of Robustness and Agreement of Quantitative Imaging Features. Tomography. 2016; 2:430–37. https://doi.org/10.18383/j.tom.2016.00235 PMID:28149958
