# Supplementary material for: A COVID-19 risk score combining chest CT radiomics and clinical characteristics to differentiate COVID-19 pneumonia from other viral pneumonias
Source: Aging (Albany NY). 2021 Mar 13;13(7):9186–224. doi: 10.18632/aging.202735 (PMC8064216; doi:10.18632/aging.202735)
Supplement: Supplementary Figures [file aging-13-202735-s002.pdf]

SUPPLEMENTARY FIGURES

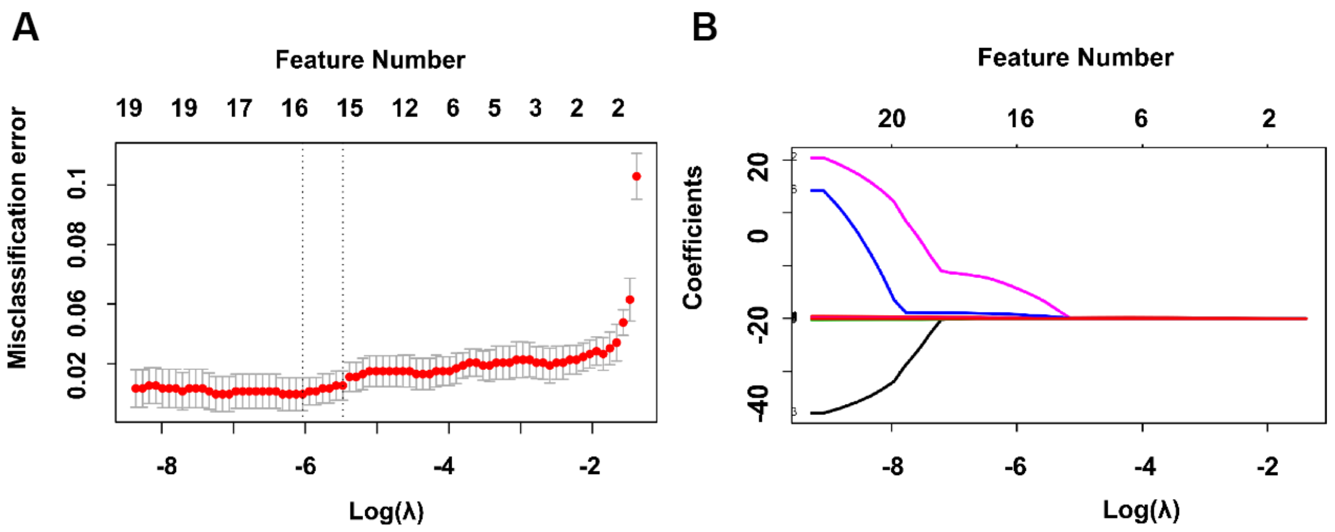

Supplementary Figure 1. The LASSO process to select the radiomic features for the construction of the patient-based risk score using radiomic features only. (**A**) The radiomic features selection procedure using LASSO regression method. To determine the best features combination for building the risk score, the control parameter λ value in the LASSO model was selected via 4-fold cross-validation with minimum criteria. The x-axis is the value of log (λ) and the y-axis is the binominal deviance in the 4-fold cross validation method 100 times. The upper x-axis is the number of non-zero-coefficient features with a given λ. The red curve indicated the average binominal deviance value with the vertical bars showing the upper and lower boundaries. The left vertical dotted line defined the λ with the least binomial deviance. The right vertical dotted line indicates the largest value of λ such that the binominal deviance is within one standard error of the minimum binominal deviance. (**B**) The LASSO coefficient profiles of the radiomic features. The figure shows the feature coefficient change with the tuning of λ value. The dotted line was plotted at the λ value determined in (**A**) resulting 16 non-zero-coefficient radiomic features.

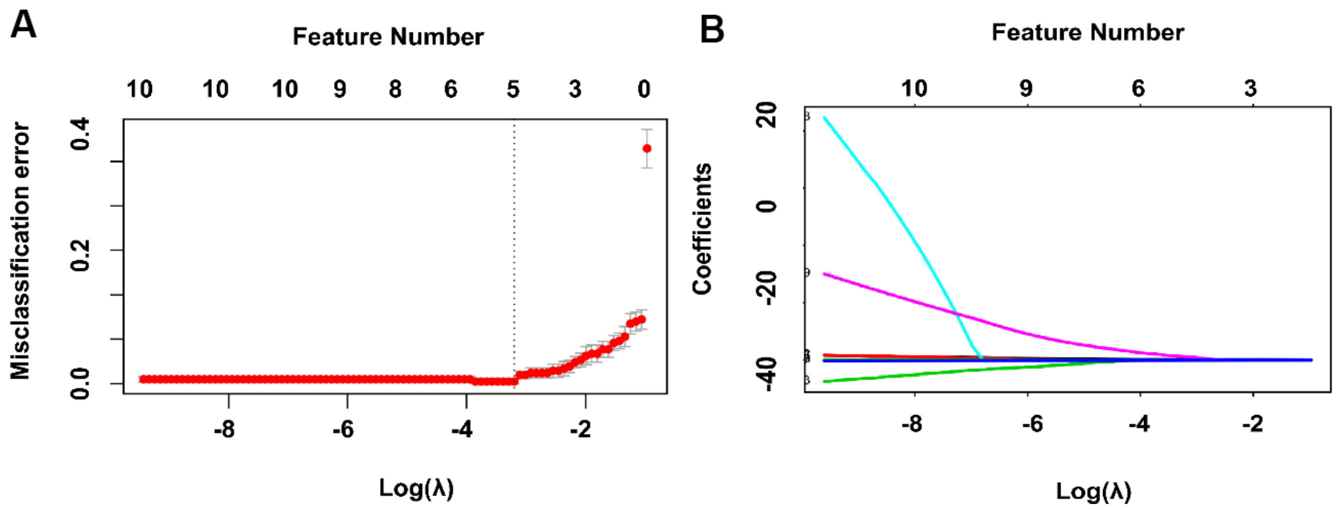

Supplementary Figure 2. The LASSO process to select the radiomics features and clinical variables for the construction of the patient-based risk score combining radiomic features and clinical variables. (**A**) The radiomic features and clinical variables selection procedure using LASSO regression method. To determine the best features combination for building the risk score, the control parameter λ value in the LASSO model was selected via 4-fold cross-validation with minimum criteria. The x-axis is the value of log (λ) and the y-axis is the binominal deviance in the 4-fold cross validation method 100 times. The upper x-axis is the number of non-zero-coefficient features with a given λ. The red curve indicated the average binominal deviance value with the vertical bars showing the upper and lower boundaries. The left vertical dotted line defined the λ with the least binomial deviance. The right vertical dotted line indicates the largest value of λ such that the binominal deviance is within one standard error of the minimum binominal deviance. (**B**) The LASSO coefficient profiles of the 17 radiomic features and clinical variables. The figure shows the feature coefficient change with the tuning of λ value. The dotted line was plotted at the λ value determined in (**A**) resulting 5 non-zero-coefficient radiomic features and clinical variables.

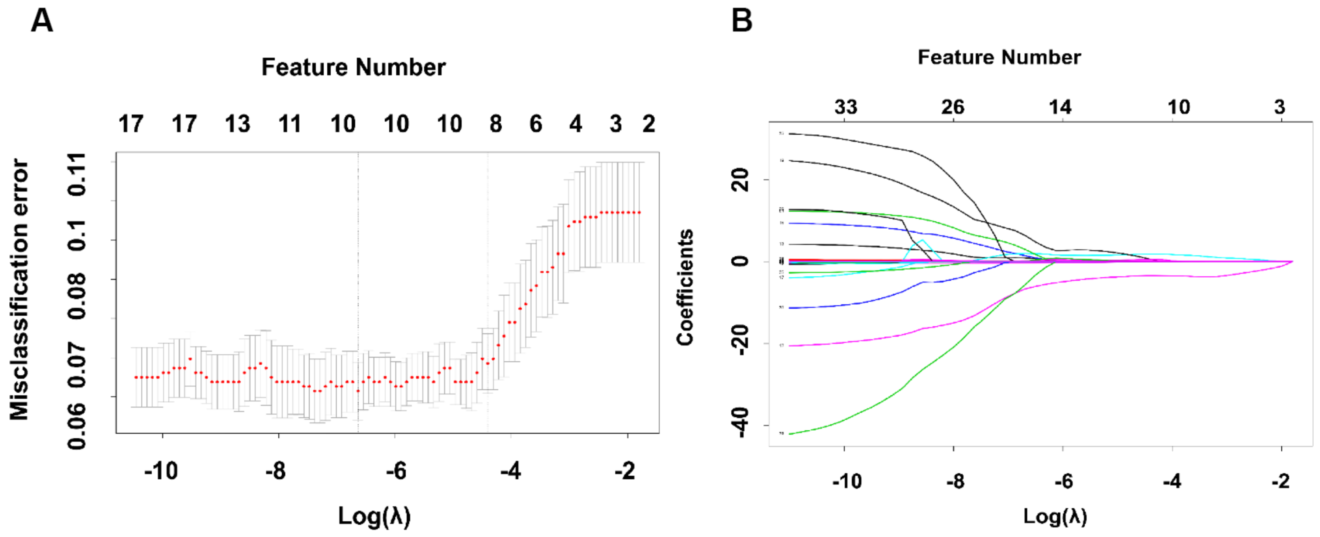

Supplementary Figure 3. The LASSO process to select the radiomic features for the construction of lesion-based risk score using radiomic features alone. (**A**) The radiomic features selection procedure using LASSO regression method. To determine the best features combination for building the risk score, the control parameter λ value in the LASSO model was selected via 4-fold cross-validation with minimum criteria. The x-axis is the value of log (λ) and the y-axis is the binominal deviance in the 4-fold cross validation method 100 times. The upper x-axis is the number of non-zero-coefficient features with a given λ. The red curve indicated the average binominal deviance value with the vertical bars showing the upper and lower boundaries. The left vertical dotted line defined the λ with the least binomial deviance. The right vertical dotted line indicates the largest value of λ such that the binominal deviance is within one standard error of the minimum binominal deviance. (**B**) The LASSO coefficient profiles of the radiomic features. The figure shows the feature coefficient change with the tuning of λ value. The dotted line was plotted at the λ value determined in (**A**) resulting the 10 non-zero-coefficient radiomic features.

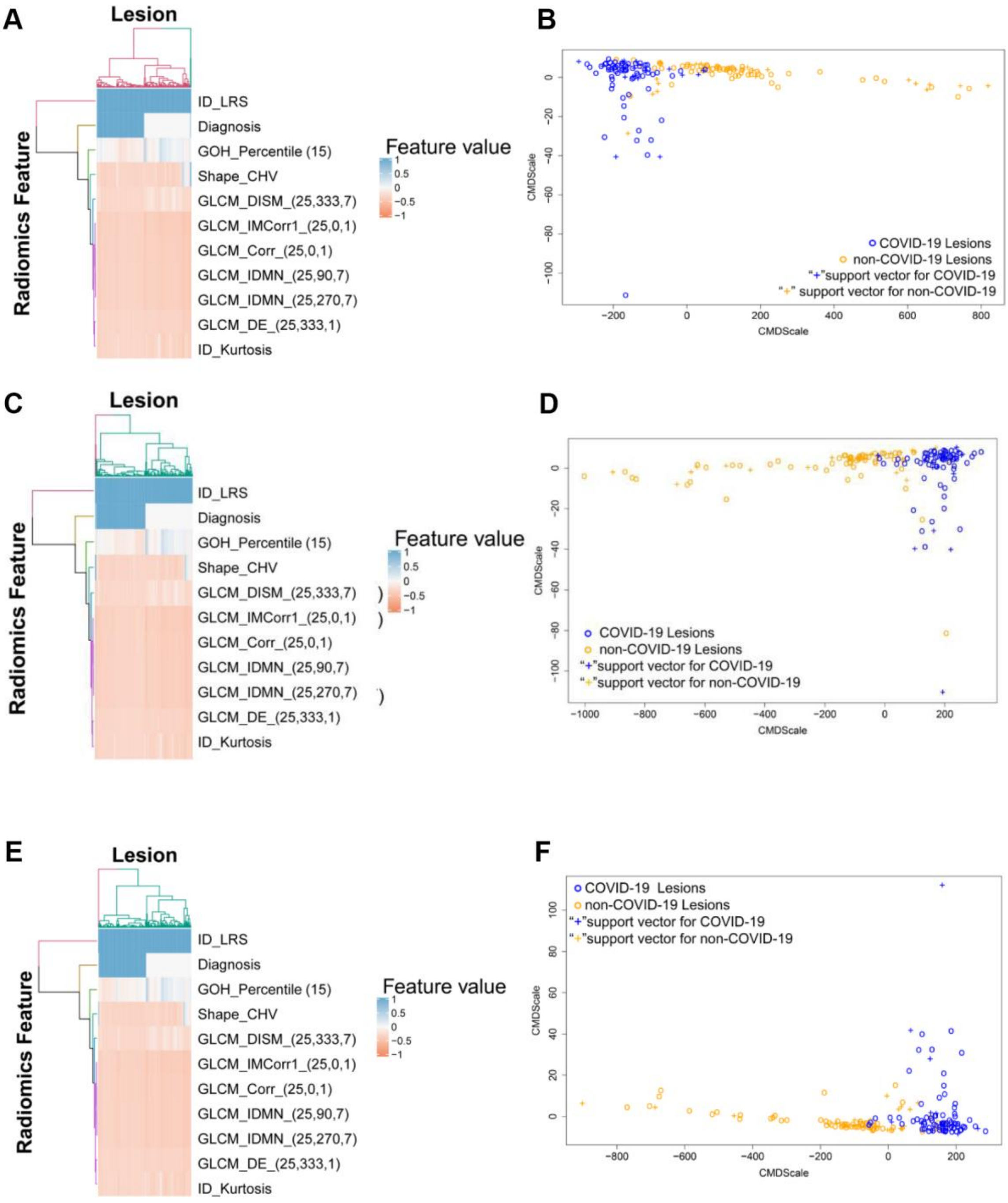
A
Lesion
Radiomics Feature
ID_LRS
Diagnosis
GOH_Percentile (15)
Shape_CHV
GLCM_DISM_(25,333,7)
GLCM_IMCorr1_(25,0,1)
GLCM_Corr_(25,0,1)
GLCM_IDMN_(25,90,7)
GLCM_IDMN_(25,270,7)
GLCM_DE_(25,333,1)
ID_Kurtosis
Feature value
B
CMDScale
CMDScale
COVID-19 Lesions
non-COVID-19 Lesions
"+"support vector for COVID-19
"+" support vector for non-COVID-19
C
D
E
F

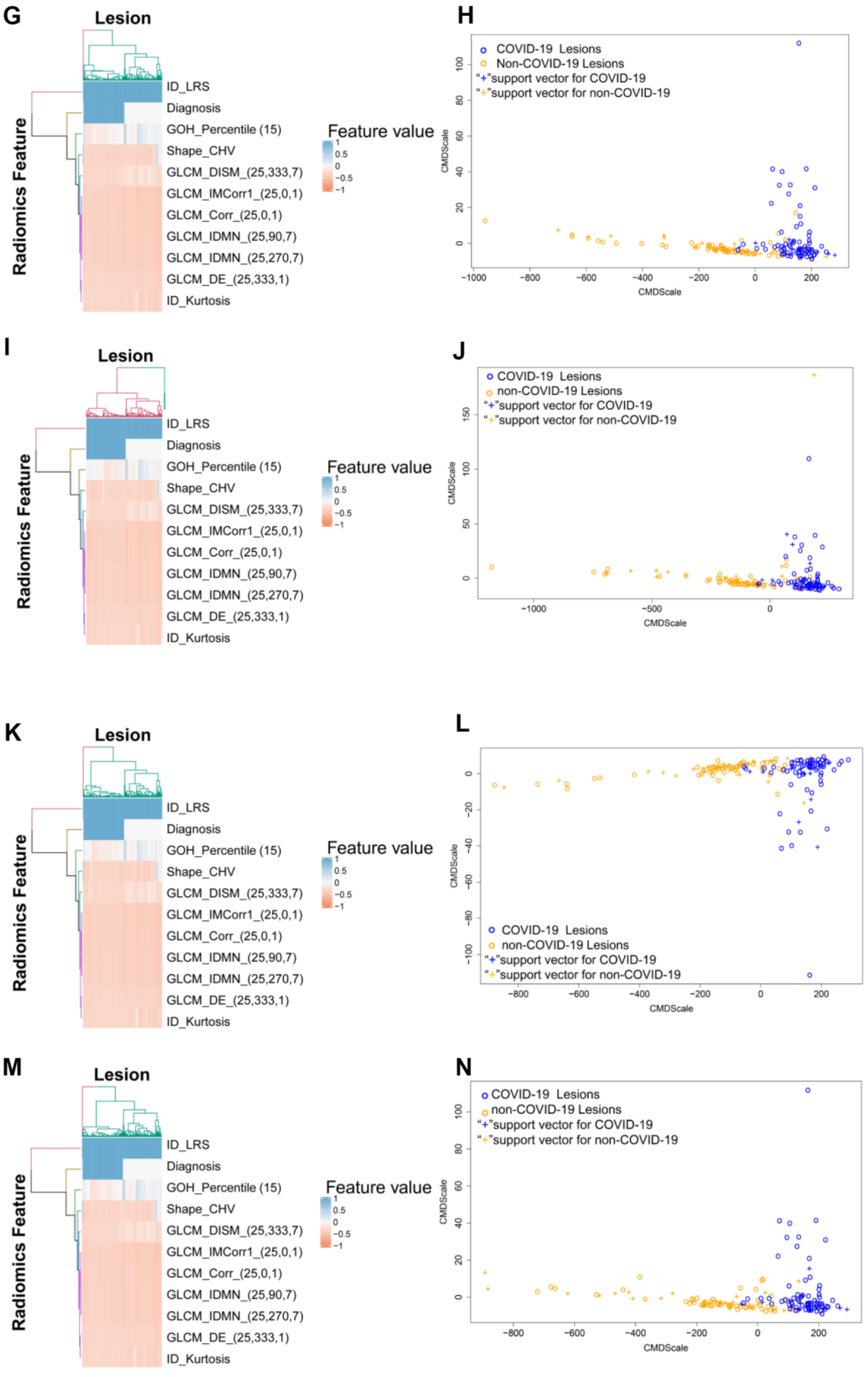

G
Lesion
Radiomics Feature
ID_LRS
Diagnosis
GOH_Percentile (15)
Shape_CHV
GLCM_DISM_(25,333,7)
GLCM_IMCorr1_(25,0,1)
GLCM_Corr_(25,0,1)
GLCM_IDMN_(25,90,7)
GLCM_IDMN_(25,270,7)
GLCM_DE_(25,333,1)
ID_Kurtosis
Feature value
H
COVID-19 Lesions
Non-COVID-19 Lesions
"+"support vector for COVID-19
"+"support vector for non-COVID-19
CMDScale
CMDScale
I
Lesion
Radiomics Feature
Feature value
J
COVID-19 Lesions
non-COVID-19 Lesions
"+"support vector for COVID-19
"+"support vector for non-COVID-19
CMDScale
CMDScale
K
Lesion
Radiomics Feature
Feature value
L
COVID-19 Lesions
non-COVID-19 Lesions
"+"support vector for COVID-19
"+"support vector for non-COVID-19
CMDScale
CMDScale
M
Lesion
Radiomics Feature
Feature value
N
COVID-19 Lesions
non-COVID-19 Lesions
"+"support vector for COVID-19
"+"support vector for non-COVID-19
CMDScale
CMDScale

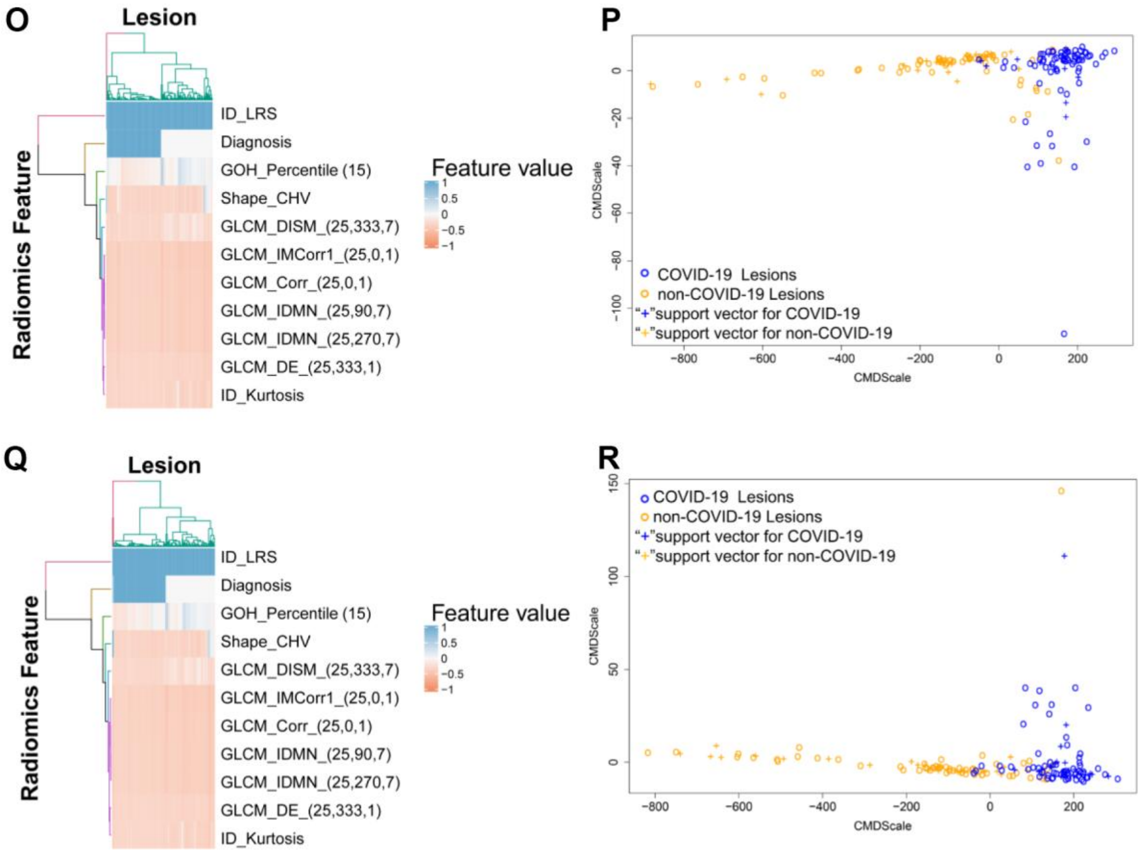

Supplementary Figure 4. The correlation of radiomics features and COVID-19 for each constituent SVM. (**A, C, E, G, I, K, M, O, Q**): The heat maps of correlation of radiomics features and COVID-19 for 9 individual constituent SVM. (**B, D, F, H, J, L, N, P, R**): The classical (metric) multidimensional scaling matrix to demonstrate the discriminative abilities of the individual constituent SVM to classify COVID-19 and non-COVID-19 using 9 constituent SVM individually. Abbreviations: SVM, support vector machine.

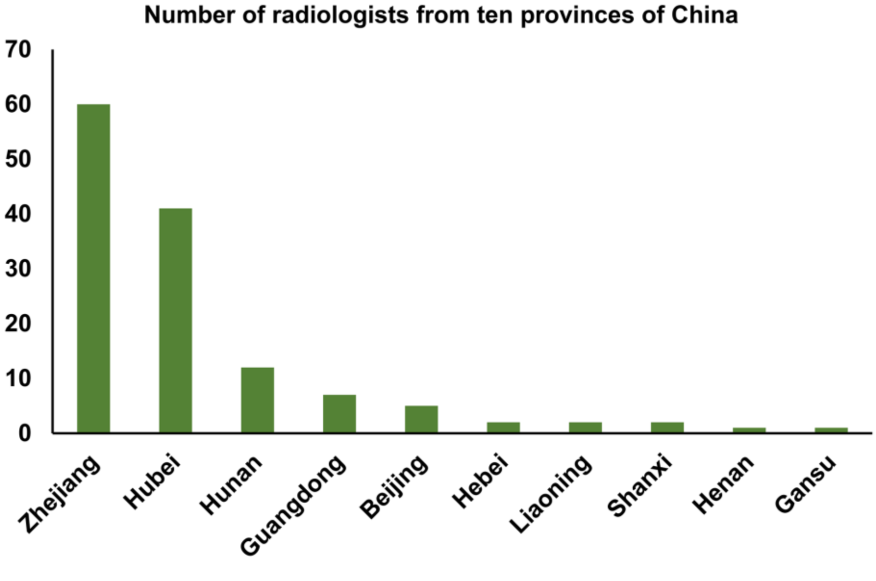

Supplementary Figure 5. The geographic distribution of 130 radiologists from 10 provinces in China including Hubei province, the epicenter of COVID-19 outbreak in China.

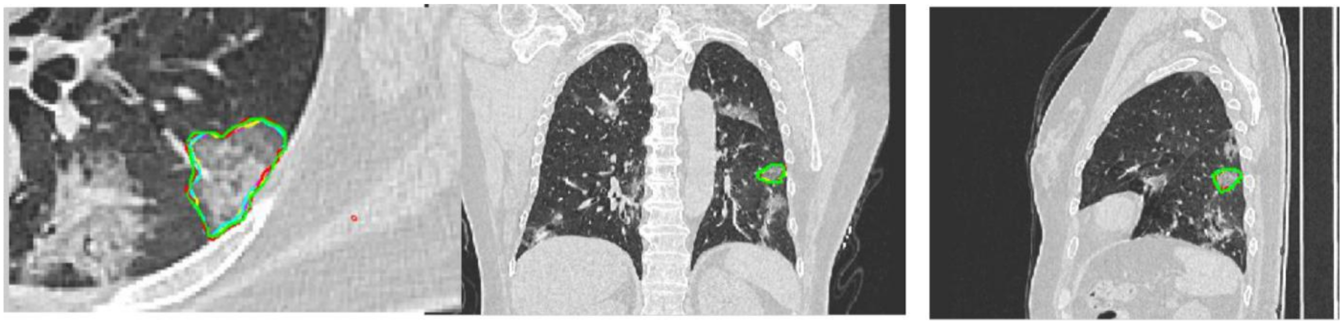

Supplementary Figure 6. Segmentations from 5 radiologists to delineate the volumes of interest in the same COVID-19 patient.
