# Supplementary material for: A COVID-19 risk score combining chest CT radiomics and clinical characteristics to differentiate COVID-19 pneumonia from other viral pneumonias
Source: Aging (Albany NY). 2021 Mar 13;13(7):9186–224. doi: 10.18632/aging.202735 (PMC8064216; doi:10.18632/aging.202735)
Supplement: Supplementary Tables 1 to 14 [file aging-13-202735-s003.pdf]

SUPPLEMENTARY TABLES

Supplementary Table 1. The 32 radiomic features selected by principal component analysis (PCA) for dimensionality reduction to construct the patient-based risk score using radiomic features only.

Radiomic features	Coefficients	Cumulative%	Explainable variance ratio%
Shape_Convex	0.913	37.116	37.116
Shape_Convex_Hull_Volume	0.926	54.347	17.231
GOH_Percentile_(15)	0.933	66.221	11.874
GOH_Percentile_(50)	0.979	75.455	9.234
GLCM_Cluster_Prominence_(25,333,4)	0.901	80.369	4.914
GLCM_Cluster_Prominence_(25,315,4)	0.972	84.221	3.852
GLCM_Cluster_Shade_(25,333,4)	0.754	86.194	1.973
GLCM_Cluster_Shade_(25,315,4)	0.829	87.825	1.631
GLCM_Cluster_Shade_(25,315,7)	0.936	89.214	1.389
GLCM_Contrast_(25,0,10)	0.689	90.605	1.391
GLCM_Contrast_(25,90,7)	0.757	91.711	1.106
GLCM_Contrast_(25,270,4)	0.837	92.582	0.871
GLCM_Correlation_(25,0,1)	0.306	93.401	0.819
GLCM_Correlation_(25,0,4)	0.954	94.124	0.723
GLCM_Correlation_(25,90,4)	0.661	94.813	0.689
GLCM_Difference_Entropy_(25,333,1)	0.396	95.446	0.633
GLCM_Dissimilarity_(25,333,7)	0.925	95.967	0.521
GLCM_Energy_(25,45,4)	0.931	96.548	0.581
GLCM_Energy_(25,45,7)	0.486	96.985	0.437
GLCM_Energy_(25,135,4)	0.654	97.386	0.401
GLCM_Information_Measure_Corr1_(25,0,1)	0.671	97.782	0.396
GLCM_Information_Measure_Corr2_(25,315,1)	0.432	98.097	0.315
GLCM_Inverse_Diff_Moment_Norm_(25,135,4)	0.779	98.395	0.298
GLCM_Inverse_Diff_Moment_Norm_(25,225,4)	0.828	98.672	0.277
GLCM_Inverse_Diff_Moment_Norm_(25,270,7)	0.669	98.884	0.212
GLCM_Inverse_Variance_(25,225,4)	0.534	99.093	0.209
GLCM_Inverse_Variance_(25,315,4)	0.289	99.287	0.194
GLCM_Max_Probability_(25,180,1)	0.275	99.454	0.167
GLCM_Sum_Entropy_(25,180,7)	0.439	99.602	0.148
ID_Kurtosis	0.354	99.74	0.138
ID_Local_Range_Std	0.119	99.879	0.139
IH_Percentile_Area_(30)	0.387	100	0.121

The values in the brackets represent the parameters used to calculate that particular radiomic feature.

Supplementary Table 2. The 20 radiomic features plus 6 clinical variables selected by principal component analysis (PCA) for dimensionality reduction to construct the patient-based risk score combining radiomic features and clinical variables.

Radiomic features and clinical variables	Coefficients	Cumulative%	Explainable variance ratio%
Lesion number	0.866	33.623	33.623
Lactate dehydrogenase	0.829	44.588	10.965
C-reactive protein	0.756	53.795	9.207
Gender	0.932	61.926	8.131
White blood cell	0.689	69.029	7.103
D-dimers	0.832	75.902	6.873
Shape_Compactness_(2)	0.911	81.423	5.521
Shape_Convex_Hull_Volume	0.874	85.786	4.363
Shape_Orientation	0.829	88.938	3.152
Shape_Roundness	0.543	91.042	2.104
GOH_Percentile_Area_(15)	0.886	92.476	1.434
GOH_Percentile_Area_(70)	0.851	93.494	1.018
GOH_Range	0.944	94.497	1.003
GLCM_Cluster_Prominence_(25,333,4)	0.756	95.499	1.002
GLCM_Cluster_Shade_(25,333,4)	0.813	96.400	0.901
GLCM_Correlation_(25,333,1)	0.925	97.143	0.743
GLCM_Correlation_(25180,7)	0.866	97.620	0.477
GLCM_Difference_Entropy_(25,333,1)	0.862	98.081	0.461
GLCM_Information_Measure_Corr_(2,25,333,7)	0.642	98.533	0.452
GLCM_Max_Probability_(25,333,1)	0.473	98.845	0.312
GLCM_Max_Probability_(250,7)	0.455	99.078	0.233
GLRLM_Long_Run_Low_Gray_Level_Empha_(2590)	0.856	99.289	0.211
ID_Global_Max	0.865	99.498	0.209
ID_Local_Entropy_Std	0.674	99.699	0.201
ID_Percentile_(30)	0.498	99.863	0.164
IH_Percentile_(30)	0.563	100.000	0.137

The values in the brackets represent the parameters used to calculate that particular radiomic feature.

Supplementary Table 3. The 20 radiomic features selected by the Mann-Whitney U test for dimensionality reduction to construct the patient-based risk score using radiomic features only.

Radiomic feature	AUC	95% CI	P
Shape_Convex_Hull_Volume	0.704	0.664-0.808	0.000
GOH_Percentile_(15)	0.890	0.828-0.921	0.000
GOH_Percentile_(50)	0.688	0.618-0.778	0.321
GLCM_Cluster_Prominence_(25,333,4)	0.544	0.464-0.632	0.467
GLCM_Cluster_Prominence_(25,315,4)	0.509	0.458-0.613	0.218
GLCM_Cluster_Shade_(25,315,7)	0.438	0.367-0.557	0.000
GLCM_Correlation_(25,0,1)	0.853	0.803-0.903	0.000
GLCM_Contrast_(25,0,1)	0.972	0.912-0.998	0.000
GLCM_Contrast_(25,270,4)	0.781	0.713-0.872	0.000
GLCM_Difference_Entropy_(25,333,1)	0.940	0.876-0.984	0.000
GLCM_Dissimilarity_(25,333,7)	0.532	0.429-0.613	0.311
GLCM_Energy_(25,135,4)	0.557	0.465-0.604	0.231
GLCM_Energy_(25,225,4)	0.532	0.487-0.612	0.367
GLCM_Information_Measure_Corr1_(25,0,1)	0.623	0.539-0.719	0.000
GLCM_ Information_Measure_Corr2_(25,315,1)	0.567	0.509-0.633	0.000
GLCM_Inverse_Diff_Moment_Norm_(25,90,7)	0.540	0.512-0.634	0.000
GLCM_Inverse_Diff_Moment_Norm_(25,270,7)	0.677	0.621-0.759	0.000
GLCM_Sum_Entropy_(25,180,7)	0.684	0.589-0.704	0.000
ID_Kurtosis	0.734	0.633-0.811	0.000
ID_Local_Range_Std	0.976	0.909-0.994	0.216

The values in the brackets represent the parameters used to calculate that particular radiomic feature.

Supplementary Table 4. The 11 radiomic features and 6 clinical variables selected by the Mann-Whitney U test for dimensionality reduction to construct COVID-19 risk score using radiomic features and clinical variables.

Radiomic features and clinical variables	AUC	95% CI	P Value
Lesion number	0.956	0.909-0.994	0.000
Gender	0.463	0.274-0.554	0.673
White blood cell	0.394	0.369-0.551	0.421
C-reactive protein	0.674	0.593-0.751	0.000
Lactate dehydrogenase	0.887	0.804-0.915	0.000
Creatine kinase isoenzyme	0.069	0.036-0.081	0.000
Shape_Compactness_(2)	0.365	0.278-0.434	0.003
GOH_Range	0.678	0.576-0.717	0.000
GLCM_Cluster_Prominence_(25,333,4)	0.604	0.512-0.723	0.004
GLCM_Correlation_(25,333,1)	0.287	0.209-0.353	0.000
GLCM_Difference_Entropy_(25,333,1)	0.724	0.667-0.771	0.000
GLCM_MaxProbability_(250,7)	0.365	0.312-0.423	0.009
GLRLM_LRLGE_(25,90)	0.767	0.676-0.812	0.000
ID_GlobalMax	0.791	0.695-0.853	0.000
ID_Percentile_(30)	0.368	0.311-0.467	0.001
IH_Percentile_(30)	0.303	0.286-0.431	0.003
IH_Percentile_Area	0.387	0.271-0.463	0.000

The values in the brackets represent the parameters used to calculate that particular radiomic feature.

Supplementary Table 5. The 16 radiomic feature selected by the LASSO for dimensionality reduction to construct the patient-based risk score using radiomic features only.

Radiomic feature	Lambda
Intercept	1.04E-02
Shape_Convex_Hull_Volume	2.07E-01
GOH_Percentile_(15)	-8.58E-02
GOH_Percentile_(50)	-4.07E-05
GLCM_AutoCorrelation(250,1)	-3.58E-06
GLCM_ClusterProminence(25,315,4)	-2.02E-05
GLCM_ClusterShade(25,315,4)	4.63E-02
GLCM_Correlation_(25,0,1)	4.04E-01
GLCM_Difference_Entropy_(25,333,1)	-4.26E-01
GLCM_Dissimilarity_(25,333,7)	6.11E+02
GLCM_Information_Measure_Corr1_(25,0,1)	1.49E+01
GLCM_Inverse_Diff_Moment_Norm_(25,90,7)	7.88E+01
GLCM_Inverse_Diff_Moment_Norm_(25,270,7)	7.91E-13
GLCM_InverseDiffMomentNorm(25,270,4)	3.54E+00
GLCM25180_7SumEntropy	-1.83E-01
ID_Kurtosis	3.45E-02
ID_Local_Range_Std	1.04E-02

The values in the brackets represent the parameters used to calculate that particular radiomic feature.

Supplementary Table 6. The 2 radiomic features and 3 clinical variables selected by the LASSO for dimensionality reduction to construct COVID-19 risk score using radiomic features and clinical variables.

Radiomic features and clinical variables	Lambda
Intercept	-1.088064e+00
Lesion number	3.450725e-01
Lactate dehydrogenase	4.697555e-01
Creatine kinase isoenzyme	3.636614e-05
GLRLM_LRLGE_(25,90)	5.842366e-03
ID_GlobalMax	-1.605967e-01

The values in the brackets represent the parameters used to calculate that particular radiomic feature.

Supplementary Table 7. The 2 radiomic features selected by the multivariable logistic regression to construct the patient-based risk score using radiomic features only in the training set.

	Coef	S.E.	Wald Z	Pr(>|Z|)	AUC	95% CI
Intercept	-3.785	27.834	-4.25	0.898	..	..
GLRLM_LRLGE_(25, 90)	19.563	175.031	7.27	<0.0001	0.813	0.698-0.876
ID_Global_Max	0.002	0.017	0.14	0.002	0.853	0.668-0.814

The values in the brackets represent the parameters used to calculate that particular radiomic feature.

Supplementary Table 8. The 2 radiomic features and 3 clinical variables used by the multivariable logistic regression to construct the COVID-19 risk score using radiomic features and clinical variables in training set.

	Coef	S.E.	Wald Z	Pr(>|Z|)	AUC	95% CI
Intercept	-114.053	559.175	-0.11	0.765	..	..
Lesion number	9.311	31.132	9.59	<0.0001	0.823	0.793-0.906
GLRLM_LRLGE_(25, 90)	122.045	687.176	2.14	<0.0001	0.718	0.624-0.845
ID_Global_Max	0.0196	0.045	3.08	0.001	0.786	0.667-0.822
Lactate dehydrogenase	0.334	2.063	7.21	<0.0001	0.861	0.779-0.916
creatine kinase isoenzymes	-7.593	38.179	-6.23	<0.0001	0.856	0.793-0.991

The values in the brackets represent the parameters used to calculate that particular radiomic feature.

Supplementary Table 9. The 3 clinical variables used by the multivariable logistic regression to construct the patient-based risk score using clinical variables only in the training set.

	Coef	S.E.	Wald Z	Pr(>|Z|)	AUC	95% CI
Intercept	-15.680	134.812	-8.13	0.898	..	..
Lesion number	2.833	17.532	2.31	<0.0001	0.812	0.749-0.917
Lactate dehydrogenase	0.104	1.337	1.19	<0.0001	0.857	0.813-0.906
creatine kinase isoenzymes	-1.674	11.294	-1.76	<0.0001	0.844	0.794-0.974

Supplementary Table 10. The 32 radiomics features selected by principal component analysis (PCA) for dimensionality reduction to construct lesion-based risk score using radiomic features only.

Radiomic Features	Coefficients	Cumulative%	Explainable variance ratio%
Shape_Convex	0.919	36.680	36.680
Shape_Convex_Hull_Volume	0.977	19.417	56.098
GOH_Percentile_(15)	0.867	10.496	66.593
GOH_Percentile_(50)	0.867	9.244	75.837
GLCM_Cluster_Prominence_(25,333,4)	0.912	4.470	80.307
GLCM_Cluster_Prominence_(25,315,4)	0.858	2.653	82.961
GLCM_Cluster_Shade_(25,333,4)	0.673	1.839	84.800
GLCM_Cluster_Shade_(25,315,4)	0.895	1.568	86.368
GLCM_Cluster_Shade_(25,315,7)	0.920	1.319	87.687
GLCM_Contrast_(25,0,10)	0.494	1.255	88.942
GLCM_Contrast_(25,90,7)	0.695	1.161	90.103
GLCM_Contrast_(25,270,4)	0.817	1.012	91.116
GLCM_Correlation_(25,0,1)	0.208	0.922	92.038
GLCM_Correlation_(25,0,4)	0.850	0.844	92.882
GLCM_Correlation_(25,90,4)	0.659	0.785	93.667
GLCM_Difference_Entropy_(25,333,1)	0.569	0.734	94.401
GLCM_Dissimilarity_(25,333,7)	0.819	0.686	95.087
GLCM_Energy_(25,45,4)	0.878	0.658	95.745
GLCM_Energy_(25,45,7)	0.401	0.647	96.392
GLCM_Energy_(25,135,4)	0.418	0.634	97.026
GLCM_Information_Measure_Corr1_(25,0,1)	0.618	0.595	97.621
GLCM_Information_Measure_Corr2_(25,315,1)	0.412	0.536	98.157
GLCM_Inverse_Diff_Moment_Norm_(25,135,4)	0.468	0.515	98.673
GLCM_Inverse_Diff_Moment_Norm_(25,225,4)	0.781	0.383	99.056
GLCM_Inverse_Diff_Moment_Norm_(25,270,7)	0.676	0.132	99.189
GLCM_Inverse_Variance_(25,225,4)	0.476	0.107	99.295
GLCM_Inverse_Variance_(25,315,4)	0.178	0.093	99.388
GLCM_Max_Probability_(25,180,1)	0.211	0.086	99.474
GLCM_Sum_Entropy_(25,180,7)	0.365	0.081	99.555
ID_Kurtosis	0.313	0.064	99.620
ID_Local_Range_Std	0.121	0.045	99.665
IH_Percentile_Area_(30)	0.371	0.043	99.708

The values in the brackets represent the parameters used to calculate that particular radiomic feature.

Supplementary Table 11. The 20 radiomics features selected by the Mann-Whitney U test for dimensionality reduction to construct the lesion-based risk score using radiomic features only.

Radiomic feature	AUC	95% CI	P
Shape_Convex_Hull_Volume	0.715	0.618-0.757	0.000
GOH_Percentile_(15)	0.837	0.768-0.954	0.000
GOH_Percentile_(50)	0.669	0.607-0.779	0.172
GLCM_Cluster_Prominence_(25,333,4)	0.499	0.401-0.681	0.305
GLCM_Cluster_Prominence_(25,315,4)	0.480	0.396-0.557	0.172
GLCM_Cluster_Shade_(25,315,7)	0.441	0.348-0.567	0.000
GLCM_Correlation_(25,0,1)	0.809	0.731-0.898	0.001
GLCM_Contrast_(25,0,1)	0.884	0.715-0.918	0.000
GLCM_Contrast_(25,270,4)	0.713	0.647-0.862	0.003
GLCM_Difference_Entropy_(25,333,1)	0.837	0.778-0.925	0.000
GLCM_Dissimilarity_(25,333,7)	0.493	0.418-0.553	0.551
GLCM_Energy_(25,135,4)	0.511	0.449-0.654	0.106
GLCM_Energy_(25,225,4)	0.474	0.388-0.591	0.493
GLCM_Information_Measure_Corr1_(25,0,1)	0.570	0.469-0.670	0.001
GLCM_ Information_Measure_Corr2_(25,315,1)	0.529	0.844-0.635	0.000
GLCM_Inverse_Diff_Moment_Norm_(25,90,7)	0.542	0.684-0.627	0.000
GLCM_Inverse_Diff_Moment_Norm_(25,270,7)	0.712	0.667-0.749	0.001
GLCM_Sum_Entropy_(25,180,7)	0.732	0.675-0.138	0.000
ID_Kurtosis	0.714	0.677-0.845	0.000
ID_Local_Range_Std	0.932	0.889-0.989	0.305

The values in the brackets represent the parameters used to calculate that particular radiomic feature.

Supplementary Table 12. The 10 radiomic features selected by the LASSO for dimensionality reduction to construct the lesion-based risk score using radiomic features only.

Radiomic feature	Lambda
Intercept	-5.023
Shape_Convex_Hull_Volume	0.006
GOH_Percentile_(15)	0.011
GLCM_Correlation_(25,0,1)	-3.858
GLCM_Difference_Entropy_(25,333,1)	1.916
GLCM_Dissimilarity_(25,333,7)	-0.080
GLCM_Information_Measure_Corr1_(25,0,1)	0.595
GLCM_Inverse_Diff_Moment_Norm_(25,90,7)	0.498
GLCM_Inverse_Diff_Moment_Norm_(25,270,7)	0.001
ID_Kurtosis	0.035
ID_Local_Range_Std	0.004

The values in the brackets represent the parameters used to calculate that particular radiomic feature.

Supplementary Table 13. The 3 radiomic features used by the multivariable logistic regression to construct the lesion-based risk score using radiomic features only.

	Coefficient	Standard error	Wald Z	Pr(>|Z|)	AUC	95% CI
Intercept	-55.389	62.895	-0.976	0.413	-	-
GLCM_Correlation_(25, 0, 1)	-6.769	5.811	-5.023	<0.0001	0.718	0.665-0.874
ID_Local_Range_Std	0.033	0.002	3112	<0.0001	0.753	0.633-0.819
GOH_Percentile_(15)	0.136	0.052	4.153	0.002	0.798	0.771-0.804

Supplementary Table 14. The diagnostic performance of individual 9 constituent SVMs.

9 Individual constituent SVM	Validation dataset	Precision	Recall	F1	AUC	95% CI	P value
SUB-SVM1	G1	1.000	0.846	0.917	0.909	0.895-0.923	
SUB-SVM2	G2	0.952	1.000	0.976	0.963	0.934-0.978	
SUB-SVM3	G3	1.000	0.833	0.909	0.9	0.892-0.912	
SUB-SVM4	G4	1.000	0.871	0.931	0.889	0.878-0.892	
SUB-SVM5	G5	1.000	0.839	0.912	0.96	0.943-0.965	
SUB-SVM6	G6	1.000	0.867	0.929	0.937	0.923-0.945	
SUB-SVM7	G7	1.000	0.893	0.943	0.944	0.954-0.962	
SUB-SVM8	G8	1.000	0.906	0.951	0.933	0.925-0.943	
SUB-SVM9	G9	1.000	0.879	0.935	0.929	0.904-0.937	
SUB-SVM-average	AVERAGE	0.995	0.882	0.934	0.929	0.912-0.934	0.231
SVM	TOTAL	0.962	0.979	0.970	0.882	0.823-0.915	
Model-G1	TOTAL	1.000	0.862	0.926	0.920	0.907-0.933	
Model-G2	TOTAL	1.000	0.877	0.935	0.930	0.918-0.943	
Model-G3	TOTAL	1.000	0.867	0.929	0.924	0.913-0.938	
Model-G4	TOTAL	1.000	0.868	0.930	0.924	0.912-0.937	
Model-G5	TOTAL	1.000	0.872	0.932	0.927	0.914-0.939	
Model-G6	TOTAL	1.000	0.873	0.932	0.928	0.915-0.940	
Model-G7	TOTAL	1.000	0.873	0.932	0.928	0.915-0.940	
Model-G8	TOTAL	1.000	0.869	0.930	0.925	0.912-0.938	
Model-G9	TOTAL	1.000	0.875	0.933	0.923	0.906-0.940	
Model-G-average	AVERAGE	1.000	0.871	0.931	0.925	0.912-0.939	0.226
WSVM	TOTAL	0.999	0.884	0.968	0.958	0.943-0.967	0.000

The COVID-19 lesion groups was randomly decomposed into 9 partitions, and all the non-COVID-19 lesions were combined with each partition of COVID-19 lesions to form an individual subset (i.e., group1 (G1) –group9 (G9)).
P values: P = 0.231 represents that there are no significant differences between SUB-SVM$_i$ (i=1-9); P = 0.226 represents that there are no significant differences between Model-G$_i$ (i=1-9); P = 0.000 represents that there are significant differences between SUB-SVM-average, Model-G-average and WSVM.
Abbreviations: WSVM, weighted support vector machine.
