# Supplementary material for: A COVID-19 risk score combining chest CT radiomics and clinical characteristics to differentiate COVID-19 pneumonia from other viral pneumonias
Source: Aging (Albany NY). 2021 Mar 13;13(7):9186–224. doi: 10.18632/aging.202735 (PMC8064216; doi:10.18632/aging.202735)
Supplement: Supplementary Table 15 [file aging-13-202735-s004.docx]

**Supplementary Table 15. The comparison of the variation of contours by different radiologists and its impact on the calculation of COVID-19 risk score using radiomic feature only (patient based analysis).**

|  |  |  |  | GLRLM_LRLGE_(25,90) | | ID_Global_Max | | COVID-19 Risk points |
| --- | --- | --- | --- | --- | --- | --- | --- | --- |
| Tools |  | Volume | Surface Area | Value | 19.563 × GLRLM_LRLGLE (25, 90) | Value | 0.002 × ID_Global_Max | −3.78 5+ 19.563 × GLRLM_LRLGLE (25, 90) + 0.002 × ID_Global_Max |
| IBEX | VOI_R_1 | 7.60116148 | 29.78872958 | 0.002823098 | 0.055152 | 2566.00 | 5.132 | 1.402152 |
|  | VOI_R_2 | 7.380523205 | 28.66423369 | 0.002810314 | 0.054902 | 2507.00 | 5.014 | 1.283902 |
|  | VOI_R_3 | 7.557709694 | 29.39070425 | 0.002793965 | 0.054583 | 2563.00 | 5.126 | 1.395583 |
|  | VOI_R_4 | 7.671166897 | 30.00937155 | 0.002898847 | 0.056632 | 2563.00 | 5.126 | 1.397632 |
|  | VOI_R_5 | 7.785590172 | 30.39869937 | 0.003101329 | 0.060588 | 2563.00 | 5.126 | 1.401588 |
|  | Mean | 7.599230289 | 29.65034769 | 0.00288551 | 0.056371 | 2552.40 | 5.1048 | 1.376171 |
|  | Std. Dev. | 0.122110168 | 0.539739667 | 0.000103832 | 0.002484337 | 20.75 | 0.050825191 | 0.051652496 |
| Pyradiomics | VOI_R_1 | 7.601 | 29.789 | 0.00282 | 0.055092 | 2566.00 | 5.132 | 1.402092 |
|  | VOI_R_2 | 7.381 | 28.664 | 0.00281 | 0.054896 | 2507.00 | 5.014 | 1.283896 |
|  | VOI_R_3 | 7.558 | 29.391 | 0.00279 | 0.054505 | 2563.00 | 5.126 | 1.395505 |
|  | VOI_R_4 | 7.671 | 30.009 | 0.00290 | 0.056654 | 2563.00 | 5.126 | 1.397654 |
|  | VOI_R_5 | 7.786 | 30.399 | 0.00310 | 0.060562 | 2563.00 | 5.126 | 1.401562 |
|  | Mean | 7.599 | 29.650 | 0.003 | 0.058608 | 2552.40 | 5.1048 | 1.378408 |
|  | Std. Dev. | 0.134 | 0.591 | 0.000 | 0.002496 | 22.73 | 0.050825 | 0.05164 |
| In-house MATLAB code | VOI_R_1 | 7.60116 | 29.78873 | 0.00282 | 0.055092 | 2566.00000 | 5.132 | 1.402092 |
|  | VOI_R_2 | 7.38052 | 28.66423 | 0.00281 | 0.054896 | 2507.00000 | 5.014 | 1.283896 |
|  | VOI_R_3 | 7.55771 | 29.39070 | 0.00279 | 0.054505 | 2563.00000 | 5.126 | 1.395505 |
|  | VOI_R_4 | 7.67117 | 30.00937 | 0.00290 | 0.056654 | 2563.00000 | 5.126 | 1.397654 |
|  | VOI_R_5 | 7.78559 | 30.39870 | 0.00310 | 0.060562 | 2563.00000 | 5.126 | 1.401562 |
|  | Mean | 7.59923 | 29.65035 | 0.00289 | 0.056459 | 2552.40000 | 5.1048 | 1.376259 |
|  | Std. Dev. | 0.13376 | 0.59126 | 0.00011 | 0.002496 | 22.73 | 0.050825 | 0.05164 |
