# Supplementary material for: A COVID-19 risk score combining chest CT radiomics and clinical characteristics to differentiate COVID-19 pneumonia from other viral pneumonias
Source: Aging (Albany NY). 2021 Mar 13;13(7):9186–224. doi: 10.18632/aging.202735 (PMC8064216; doi:10.18632/aging.202735)
Supplement: Supplementary Table 16 [file aging-13-202735-s005.docx]

**Supplementary Table 16. The comparison of the variation of contours by different radiologists and its impact on the calculation of COVID-19 risk score using radiomic feature only (lesion based analysis).**

| Tools |  | Volume | Surface Area | GOH_Perrcentile(15) | | GLCM_Correlation_(25,0,1) | | ID_Local_Range_Std | | COVID-19 Risk Score |
| --- | --- | --- | --- | --- | --- | --- | --- | --- | --- | --- |
|  |  |  |  | VALUE | 0.136×GOH_Percentile_(15) | VALUE | −6.769×GLCM_Correlation_(25,0,1) | VALUE | 0.33×ID_Local_Range_Std | −55.389+0.136×GOH_Percentile_(15) −6.769×GLCM_Correlation_(25,0,1)+ 0.033×ID_Local_Range_Std |
| IBEX | VOI_R_1 | 7.60116148 | 29.78872958 | 27.64055031 | 0.765019848 | 0.765019848 | 282.9975291 | 282.9975291 | 93.38918 | 34.9903 |
|  | VOI_R_2 | 7.380523205 | 28.66423369 | 27.96157074 | 0.736942978 | 0.736942978 | 299.604088 | 299.604088 | 98.86935 | 40.51412 |
|  | VOI_R_3 | 7.557709694 | 29.39070425 | 27.7712069 | 0.753244703 | 0.753244703 | 286.9724246 | 286.9724246 | 94.7009 | 36.31978 |
|  | VOI_R_4 | 7.671166897 | 30.00937155 | 28.22079126 | 0.755159522 | 0.755159522 | 297.6989717 | 297.6989717 | 98.24066 | 39.92069 |
|  | VOI_R_5 | 7.785590172 | 30.39869937 | 28.07342047 | 0.765072316 | 0.765072316 | 294.6415521 | 294.6415521 | 97.23171 | 38.8917 |
|  | Mean | 7.599230289 | 29.65034769 | 27.93350794 | 0.755087873 | 0.755087874 | 292.3829131 | 292.3829131 | 96.48636132 | 38.12732 |
|  | Std. Dev. | 0.122110168 | 0.539739667 | 0.189323596 | 0.011521424 | 0.009407203 | 7.121553811 | 5.814724317 | 2.350112758 | 2.377254346 |
| Pyradiomics | VOI_R_1 | 7.601 | 29.789 | 27.641 | 18.308 | 18.308 | 282.998 | 282.998 | 93.38934 | 34.99052 |
|  | VOI_R_2 | 7.381 | 28.664 | 27.962 | 17.636 | 17.636 | 299.604 | 299.604 | 98.86932 | 40.51415 |
|  | VOI_R_3 | 7.558 | 29.391 | 27.771 | 18.026 | 18.026 | 286.972 | 286.972 | 94.70076 | 36.31962 |
|  | VOI_R_4 | 7.671 | 30.009 | 28.221 | 18.072 | 18.072 | 297.699 | 297.699 | 98.24067 | 39.92073 |
|  | VOI_R_5 | 7.786 | 30.399 | 28.073 | 18.309 | 18.309 | 294.642 | 294.642 | 97.23186 | 38.89179 |
|  | Mean | 7.599 | 29.650 | 27.934 | 18.0702 | 18.071 | 292.383 | 292.383 | 96.48639 | 38.1273596 |
|  | Std. Dev | 0.134 | 0.591 | 0.207 | 0.275708542 | 0.247 | 7.121497806 | 6.370 | 2.350094276 | 2.377236656 |
| In-house  MATLAB code | VOI_R_1 | 7.60116 | 29.78873 | 27.64055 | 18.3082 | 18.30820 | 282.99753 | 282.99753 | 93.38918 | 34.9903 |
|  | VOI_R_2 | 7.38052 | 28.66423 | 27.96157 | 17.63627 | 17.63627 | 299.60409 | 299.60409 | 98.86935 | 40.51412 |
|  | VOI_R_3 | 7.55771 | 29.39070 | 27.77121 | 18.0264 | 18.02640 | 286.97242 | 286.97242 | 94.7009 | 36.31978 |
|  | VOI_R_4 | 7.67117 | 30.00937 | 28.22079 | 18.07222 | 18.07222 | 297.69897 | 297.69897 | 98.24066 | 39.92069 |
|  | VOI_R_5 | 7.78559 | 30.39870 | 28.07342 | 18.30945 | 18.30945 | 294.64155 | 294.64155 | 97.23171 | 38.8917 |
|  | Mean | 7.59923 | 29.65035 | 27.93351 | 18.070508 | 18.07051 | 292.382912 | 292.38291 | 96.48636096 | 38.12731805 |
|  | Std. Dev. | 0.13376 | 0.59126 | 0.20739 | 0.275727154 | 0.24662 | 7.121554412 | 6.36971 | 2.350112956 | 2.377254412 |

Abbreviation: VOI_R_i, the VOI contoured by the ith radiologist; Std. Dev., standard deviation
